# Improving EEG Forward Modeling Using High-Resolution Five-Layer BEM-FMM Head Models: Effect on Source Reconstruction Accuracy

**DOI:** 10.3390/bioengineering11111071

**Published:** 2024-10-26

**Authors:** Guillermo Nuñez Ponasso, William A. Wartman, Ryan C. McSweeney, Peiyao Lai, Jens Haueisen, Burkhard Maess, Thomas R. Knösche, Konstantin Weise, Gregory M. Noetscher, Tommi Raij, Sergey N. Makaroff

**Affiliations:** 1 Department of Electrical and Computer Engineering, Worcester Polytechnic Institute, Worcester, MA 01609, USA; wawartman@wpi.edu (W.A.W.); plai@wpi.edu (P.L.);; 2 Institute of Biomedical Engineering and Informatics, Technische Universität Ilmenau, 98693 Ilmenau, Germany; 3 Max Plank Insititute for Human Cognitive and Brain Sciences, 04103 Leipzig, Germany; maess@cbs.mpg.de (B.M.); kweise@cbs.mpg.de (K.W.); 4 Athinoula A. Martinos Center for Biomedical Imaging, Massachusetts General Hospital, Harvard Medical School, Boston, MA 02129, USA

**Keywords:** electroencephalography (EEG), EEG source analysis, EEG dipole reconstruction, brain imaging, boundary element method (BEM), fast multipole method (FMM), adaptative mesh refinement (AMR)

## Abstract

Electroencephalographic (EEG) source localization is a fundamental tool for clinical diagnoses and brain-computer interfaces. We investigate the impact of model complexity on reconstruction accuracy by comparing the widely used three-layer boundary element method (BEM) as an inverse method against a five-layer BEM accelerated by the fast multipole method (BEM-FMM) and coupled with adaptive mesh refinement (AMR) as forward solver. Modern BEM-FMM with AMR can solve high-resolution multi-tissue models efficiently and accurately. We generated noiseless 256-channel EEG data from 15 subjects in the Connectome Young Adult dataset, using four anatomically relevant dipole positions, three conductivity sets, and two head segmentations; we mapped localization errors across the entire grey matter from 4000 dipole positions. The average location error among our four selected dipoles is ∼5mm (±2mm) with an orientation error of ∼$12^{\circ }$ (±$7^{\circ }$). The average source localization error across the entire grey matter is ∼9mm (±4mm), with a tendency for smaller errors on the occipital lobe. Our findings indicate that while three-layer models are robust under noiseless conditions, substantial localization errors (10–20mm) are common. Therefore, models of five or more layers may be needed for accurate source reconstruction in critical applications involving noisy EEG data.

## 1. Introduction

EEG source reconstruction, or source localization, involves estimating the source location of neural activity within the brain from EEG-recorded measurements [1]. Applications include pre-surgical screening for the treatment of drug-resistant epilepsy [2], brain-computer interfaces (BCIs) [3], and the analysis of event-related potentials in a wide range of scenarios [4,5,6]. Due to the large susceptibility of EEG to noise and the ill-posedness of the EEG inverse problem [7], it is important to reduce modeling errors in the forward step of source localization pipelines; this can be carried out by distinguishing the relevant regions of different conductivity present in the head [8,9], utilizing accurate values for such conductivities [10], and matching the geometry and features of the head closely using high-resolution models [11]. For example, the geometry of the intracortical compartments can be very important for source reconstruction using source models where the dipole orientation is perpendicular to the cortex [12].

Many excellent open-source software packages implement EEG source localization, including Brainstorm [13], FieldTrip [14], MNE [15], and EEGLab [16]. These BEM implementations typically use three layers extracted from the subject’s MRI image: scalp, outer skull, and inner skull. For most practical applications, the resolution of these layers is limited to less than 10,000 triangles per layer. One of the main reasons for this limitation is that these packages use the classical potential-based formulation of the BEM [17,18,19], which involves dense system matrices and, as such, is unable to compute leadfields using large, high-resolution models. Complicating this issue is the fact that the intracortical compartments typically involve very narrow gaps [20] between the grey and white matter layers, where the sources are placed. This can cause large numerical inaccuracies in the forward computations, which need to be resolved with refinement techniques such as *adaptive mesh refinement* (AMR), also known as *h*-refinement [21,22], or the less common *p*-refinement [23], which consists of adaptively increasing the polynomial order of the local approximations of the variable of interest (potential or charge) on the mesh triangles. Both refinement techniques cause a computational overhead, which may be avoided in three-layer models as long as the sources are far away from the discretized shells.

Modern automated human-head segmentation tools (FreeSurfer, [24]; SPM12, [25]) can provide high-resolution skin, skull, cerebrospinal fluid (CSF), grey matter (GM), and white matter (WM) layers, among other tissues. The charge-based formulation of the BEM, coupled with *fast multipole method* (FMM) acceleration, or BEM-FMM [19,26], can rapidly compute forward solutions for high-resolution models involving tens of millions of facets, thereby removing the ∼10,000 triangle limitation of conventional potential-based BEM. Therefore, BEM-FMM could be used as the basis of high-resolution multi-layer EEG source reconstruction pipelines, offering a powerful alternative to high-resolution FEM-based forward solvers [27].

To assess the potential improvements in source reconstruction that may arise from the use of BEM-FMM, we consider the following question: How accurate would the three-layer BEM source reconstruction results be if we assume the high-resolution five-layer BEM-FMM models are ground truth? We investigated this in a modeling experiment for the canonical EEG inverse problem of single-dipole fitting.

There are many studies that consider the influence of different uncertainty factors on EEG source localization: head model complexity [9], conductivity uncertainty [10], and white matter anisotropy [28,29], among others. In all these studies, the finite element method (FEM) was used for forward modeling, i.e., for generating electrode voltages given a dipole source in the brain. Here, we use a high-resolution BEM-FMM to compute the forward solution. This is particularly motivated by the following reasons:

(i) Studies of dipole localization accuracy using BEM [30,31,32] are relatively less common than those using FEM [9,10,28,29,33]. The use of BEM-FMM allows us to carry out more tests and with higher resolution than conventional BEM.
(ii) BEM-FMM models have been found to be comparable or superior to FEM in terms of speed and accuracy in certain applications; for example, it has been found [34] that zero-order BEM-FMM is comparable in accuracy to second-order FEM for TMS modeling in the concentric spheres model. It was also found in [34] that zero-order BEM-FMM was the highest-accuracy method that they could implement within their computational constraints using a realistic, high-resolution brain model. This leads them to use zero-order BEM-FMM as their ground truth solution.
(iii) Prescribing arbitrary finite-length dipoles, or point dipoles—which are the most common mathematical model for the simultaneous firing of a large number of neurons [35]—is an easy task in BEM, as the method inherently allows for arbitrary incident fields anywhere in space. On the other hand, modeling dipoles in FEM is much more challenging: several approaches are available [36], but using them would involve an additional error estimation; see also the St. Venant approach used in [10,37].

With regards to (ii), we must mention that one major advantage of FEM over BEM is that FEM can easily model tissue anisotropy. Although it is possible to model anisotropy in BEM [38], most BEM/BEM-FMM implementations do not have this capability at the time of writing.

- Section 2 describes the materials used throughout the paper and the methods used for the analysis of four dipole locations chosen consistently across subjects;
- Section 3 describes a complementary analysis to the one in Section 2, where we generated error maps for source localization across the entire grey matter surface of each subject;
- Section 4 summarizes our results;
- Section 5 includes a brief discussion and interpretation.

In Supplement 1 (Supplementary Tables S1–S14), we include additional tables of results, and in Supplement 2 (Supplementary Figures S1–S17), we include additional subject error maps and figures.

## 2. Localization Error in Selected Locations Across Varying Subjects and Parameters

**Outline of methodology**: We set up the following numerical experiment, which is carried out for a total of 15 subjects, three conductivity sets, two mesh segmentations, and four dipole locations:

(i) We placed a dipole at a location, $p_{0}$, on the midsurface between the CSF-GM and GM-WM tissue interfaces of our subject with an orientation, $q_{0}$, normal to the CSF-GM interface.
(ii) We simulated a single time sample of noiseless EEG data using the charge-based formulation of the boundary element method with fast multipole method acceleration (BEM-FMM) and adaptive mesh refinement (AMR), over a high-resolution five-layer (seven-compartment) head volume conduction model.
(iii) We used the simulated EEG data to perform source reconstruction with a low-resolution three-layer head model, which is similar to the ones widely used in EEG source reconstruction. By doing so, we found the best fit for location $p_{1}$, and orientation $q_{1}$ was provided by the FieldTrip Toolbox’s [14] source localization procedures.
(iv) We computed the distance $∥p_{0}-p_{1}{∥}_{2}$ between both locations and the angle between $q_{0}$ and $q_{1}$ to measure the error of the fit.

**Five-layer models.** We used the SimNIBS package [39] to obtain two segmentations from the T1- and T2-weighted MRI data from 15 subjects of the Connectome Young Adult dataset [40]. One segmentation was obtained using the option mri2mesh, which performs segmentation based on FreeSurfer [24], and the other used headreco, which is based on SPM/CAT [25]. The FreeSurfer and Headreco segmentations generate five main layers: skin, skull, CSF, grey matter, and white matter.

Each skin, skull, and CSF mesh from the FreeSurfer segmentations contains approximately 120,000 triangles, whereas the grey and white matter meshes contain circa 250,000 triangles. Headreco segmentations comprise approximately 150,000 triangles for skin and skull, 100,000 triangles for CSF, 300,000 triangles for grey matter, and 350,000 triangles for white matter. So, in total, our high-resolution FreeSurfer models have over 1 M degrees of freedom. This number is about twice as large in the Headreco models.

Both segmentations include two additional tissue meshes; in the case of FreeSurfer, these are the cerebellum and ventricles, and for Headreco, these are the eyes and ventricles. One may argue that this should limit comparability between segmentations to some degree; however, the location of the two additional compartments is distant from most electrode positions. More details on the MRI data acquisition, spatial coregistration, segmentation, and mesh generation for this dataset can be found in [40,41].

We tested three tissue conductivity sets for each subject, which we labeled IT’IS7 [42,43], VWB7 [10,44], and SimNIBS7 [39]. The conductivity values in IT’IS7 and SimNIBS7 were determined experimentally, and the values in VWB7 were estimated from experimental conductivity values [45,46,47,48,49]. We made no attempt to estimate the individual tissue conductivities of each subject, although we mention that this may be possible using the methods of [50]. All conductivity sets for five-layer models can be found in Table 1, and for additional information on the conductivity of living tissues, see [1].

Each of the chosen conductivity sets is optimized for different scenarios: The IT’IS7 set provides conductivity values for frequencies up to 1MHz, SimNIBS7 specifies conductivities adequate for TMS and TES (see also [51,52]), and VWB7 has been derived from studies on the uncertainty of EEG source localization with respect to conductivity values.

The only set that provides conductivity values for all the layers is IT’IS7 [43]. For VWB7, no standard cerebellum conductivities are given. We chose a 40–60 % weighted average between the conductivities of grey matter and white matter as a conductivity value for the cerebellum. IT’IS7 assigns the same conductivity value as CSF for the eye’s vitreous humor. Hence, we assign the corresponding CSF conductivity for the eye layer in each Headreco conductivity set. We must remark, however, that the conductivity values of these additional layers are mostly irrelevant in the EEG problem since these are located far away from the scalp electrodes.

**Three-layer models.** From each FreeSurfer seven-compartment head model, we created decimated (downsampled) three-layer models using the software MeshLab [53]. Namely, we created three new meshes labeled SKIN, SKULL, and BRAIN from the high-resolution skin, skull, and CSF FreeSurfer layers of each subject.

To obtain these new meshes, we first apply a screened Poisson surface reconstruction filter [54], followed by a quadratic edge collapse decimation filter with 14,000 triangles as a target. After this is carried out, we apply a Taubin smoothing filter [55].

We used these three-layer meshes to carry out dipole source localization using the FieldTrip toolbox [14]. In other words, we used the three-layer model as our inverse volume conduction model. For every choice of conductivity for the forward five-layer model, we have the corresponding three-layer conductivities labeled IT’IS3, VWB3, and SimNIBS3; see Table 2. Each conductivity set was chosen to be as consistent as possible with its seven-compartment analog.

**Figure 1:**
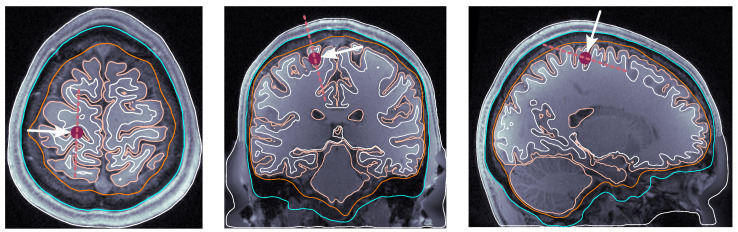
Dipole placement in the transverse, coronal, and sagittal planes, respectively, for dip1 (posterior wall of the central sulcus) in subject 110411. The maroon dot indicates the dipole position on each view. The dashed line indicates the dipole orientation, and the arrow pointing towards the dipole dot is oriented perpendicularly to the dashed line.

**Figure 2:**
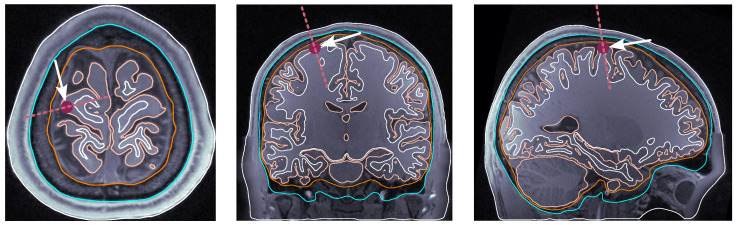
Dipole placement in the transverse, coronal, and sagittal planes, respectively, for dip2 (${M1}_{\mathrm{HAND}}$) in subject 130013. The maroon dot indicates the dipole position on each view. The dashed line indicates the dipole orientation, and the arrow pointing towards the dipole dot is oriented perpendicularly to the dashed line.

**Figure 3:**
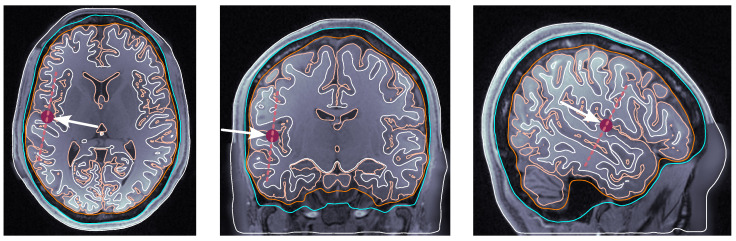
Dipole placement in the transverse, coronal, and sagittal planes, respectively, for dip3 (temporal lobe and Heschl’s gyrus) in subject 117122. The maroon dot indicates the dipole position on each view. The dashed line indicates the dipole orientation, and the arrow pointing towards the dipole dot is oriented perpendicularly to the dashed line.

**Figure 4:**
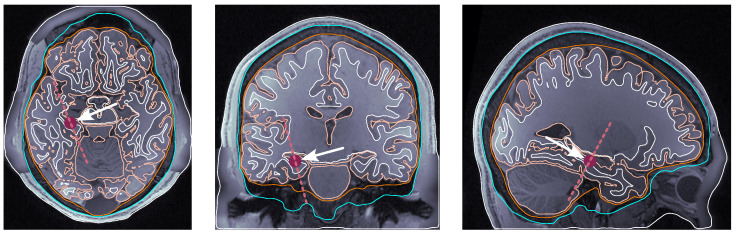
Dipole placement in the transverse, coronal, and sagittal planes, respectively, for dip4 (medio-temporal lobe) in subject 122620. The maroon dot indicates the dipole position on each view. The dashed line indicates the dipole orientation, and the arrow pointing towards the dipole dot is oriented perpendicularly to the dashed line.

**Source dipole placement.** The following are the four selected locations that we sampled consistently across all subjects:

(i) dip1 — Posterior wall of the central sulcus, somatosensory cortex, and tangential dipole; Figure 1;
(ii) dip2 — ${M1}_{\mathrm{HAND}}$ region, primary motor cortex, and radial dipole; Figure 2;
(iii) dip3 — Temporal lobe, along the Heschl’s gyri or transverse temporal gyri; Figure 3;
(iv) dip4 — Medio-temporal region; Figure 4.

The position dip3 is at the superior part of the temporal cortex and has been chosen to be studied in both auditory and language areas located at the posterior or anterior part around the same depth. The position dip4 is relevant in epileptogenic analyses [56,57].

**Figure 5:**
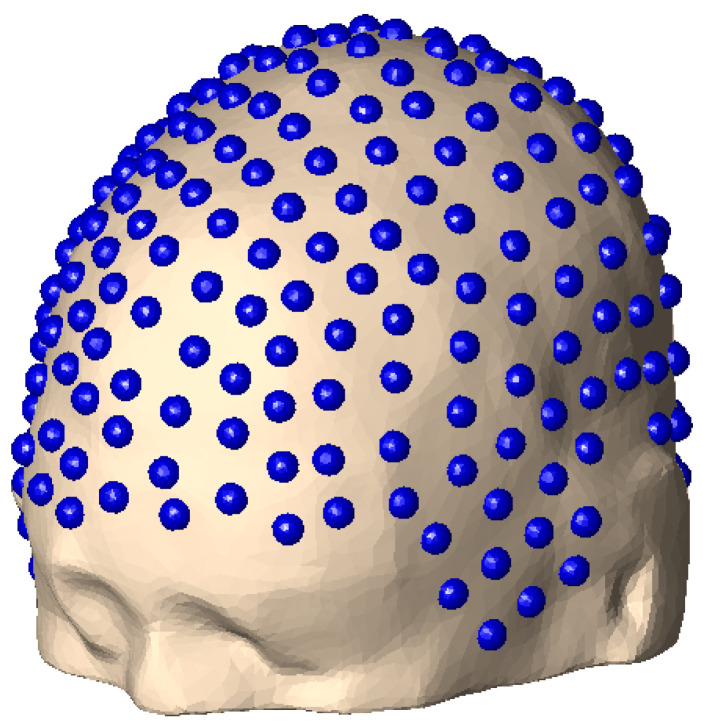
High-density EEG system of 256 electrodes placed on the skin layer of Connectome subject 110411.

**Electrode Placement:** We placed 256 electrodes in each subject’s FreeSurfer, Headreco, and decimated FreeSurfer skin meshes; see Figure 5. The high-density 256 dry electrode system that we used is described in [58]; see [59]. Since our dataset [41] does not include data on the three anatomical landmarks of the nasion, left preauricular point (LPA), or right preauricular point (RPA), we did a semi-manual electrode placement on each subject’s skin layer using the FieldTrip function ft_electrode_placement, first using the interactive option, followed by the projection option. In this way, each electrode was assigned to a triangle in the skin layer. We could then retrieve the electrode voltages from the BEM-FMM calculation of the skin potential. This was carried out by taking the value of the potential at the mesh triangle of each electrode.

**Forward solution with BEM-FMM:** We used the charge-based formulation of the BEM [60,61], together with FMM acceleration [62].

In this formulation of the BEM forward problem, the total electric field, E, is separated into two components, an impressed electric field, $E^{i}$, and a *secondary* electric field $E^{s}$, so that $E=E^{i}+E^{s}$. In our setting, the impressed field is created by a current dipole [63,64] in an infinite homogeneous medium having the conductivity of grey matter. Such a current dipole is given by an isotropic current source of strength $+I_{0}$ and an isotropic current sink of strength $-I_{0}$, both measured in amperes. For point current dipoles, the length of the displacement vector, d, from the sink to the source is small with respect to the distance of the dipole center, $r_{0}$, to the observation point, r. In this case, the impressed electric field can be approximated as $E^{i}=-\nabla \varphi^{i}$, where

(1) $$\varphi^{i}\left(r\right)=\frac{I_{0}}{4\pi \sigma_{\mathrm{GM}}}\frac{d\;\cdot \;(r-r_{0})}{|r-r_{0}{|}^{3}}.$$

Here, the constant $\sigma_{\mathrm{GM}}$ is the macroscopic conductivity of grey matter tissue. When this constant is measured in ${S\;m}^{-1}\;={A\;V}^{-1}m^{-1}\;$}, and the distances are measured in meters, then the potential $\varphi^{i}$ is measured in volts as desired.

The secondary electric field is always conservative and arises from the electric charges deposited on each surface of discontinuity, *S*, of the conductivity upon activation of the impressed electric field. To compute the secondary field, $E^{s}$, we need to calculate the surface charge density, ρ, on *S*, measured in $C\;m^{-2}\;$ An integral equation can be derived [19] using the quasi-static approximation of Maxwell’s equations and the following boundary conditions:

(2) $$\left\{\begin{array}{cc} \sigma_{+}E_{+}\;\cdot \;n=\sigma_{-}E_{-}\;\cdot \;n, & \;\mathrm{on}\;S\\ E_{-}^{i}=E_{+}^{i}, & \;\mathrm{on}\;S \end{array}\right..$$

Here, n(r) denotes the outward unit normal vector to the point r∈S, and $f_{\pm }\left(r\right)=\lim_{\epsilon \to 0}f\left(r\pm \epsilon n\left(r\right)\right)$ for all r∈S. The function σ(r) is the conductivity of our head model at an arbitrary point r in 3D space, measured in ${S\;m}^{-1}$. The resulting integral equation for ρ is given below [26,65,66]:

(3) $$\begin{array}{c} \frac{\rho (r)}{2\varepsilon_{0}}-K\left(r\right)n\left(r\right)\;\cdot \;\int_{S}\frac{\rho (r^{\prime })}{4\pi \varepsilon_{0}}\frac{r-r^{\prime }}{|r-r^{\prime }{|}^{3}}ds\left(r^{\prime }\right)\\ =K\left(r\right)E^{i}\left(r\right)\;\cdot \;n\left(r\right), \end{array}$$

where $\varepsilon_{0}\approx 8.8541\;\times \;10^{-12}\;{F\;m}^{-1}$ is the permittivity of free space, n(r) is the outward unit normal vector to *S* at r, and $K:=\left(\sigma_{-}-\sigma_{+}\right)/\left(\sigma_{-}+\sigma_{+}\right)$ is the conductivity contrast at r∈S. Since the surface element $ds(r^{\prime })$ is measured in $m^{2}$, and both the unit normal n and the conductivity contrast *K* are dimensionless quantities, all terms in Equation (3) are measured in ${V\;m}^{-1}$.

Equation (3) can be discretized using the Galerkin method. For a triangular mesh model comprising *M* facets, which approximates the geometry of each surface of discontinuity *S*, the discrete version of Equation (3) at the *m*-th facet reads

(4) $$\begin{array}{c} \frac{\rho_{m}}{2\varepsilon_{0}}-K_{m}n_{m}\;\cdot \;\sum_{n=1;\;n\neq m}^{M}\frac{\rho_{n}}{4\pi \varepsilon_{0}}\frac{r_{m}-r_{n}}{|r_{m}-r_{n}{|}^{3}}\\ =K_{m}E^{i}\left(r_{m}\right)\;\cdot \;n_{m}. \end{array}$$

Here, $\rho_{m}$, $K_{m}$, $n_{m}$, and $r_{m}$ are the charge density, conductivity contrast, outward unit normal, and triangle center at the *m*-th facet, respectively. The summation term is an *n*-body computation that we accelerated using FMM [62,67,68]. In our computations, we used the implementation of the fast multipole method provided by the FMM3D library [69]. The FMM-accelerated summation was computed to a digit precision of $1\;\times \;10^{-6}$. For reference, the typical FMM digit precision in most applications ranges from $1\;\times \;10^{-4}$ to $1\;\times \;10^{-2}$.

To estimate $\rho_{m}$ at every facet, successive approximations, $\rho_{m}^{\left(0\right)},\rho_{m}^{\left(1\right)},\cdots ,\rho_{m}^{\left(N\right)}$, of the charge density are computed using FMM acceleration. After each step, we applied the generalized residual method (GMRES) to find an approximation to $\rho_{m}$ of the form

(5) $$\rho_{m}=\alpha_{0}\rho_{m}^{\left(0\right)}+\cdots +\alpha_{N}\rho_{m}^{\left(N\right)},$$

where the scalars $\alpha_{i}$ are chosen to minimize the residual:

(6) $${\left|\frac{\rho_{m}}{2\varepsilon_{0}}-K_{m}n_{m}\;\cdot \;\left(\sum_{\begin{array}{c} n=1\\ n\neq m \end{array}}^{M}\frac{\rho_{n}}{4\pi \varepsilon_{0}}\frac{r_{m}-r_{n}}{|r_{n}-r_{m}{|}^{3}}+E^{i}\left(r_{m}\right)\right)\right|}^{2}.$$

See [19] for more details on the computation of EEG forward solutions using BEM-FMM. In our case, we stopped iterating when the GMRES residual was below $1\;\times \;10^{-5}\;V^{2}\;m^{-2}$. For reference, typical BEM-FMM GMRES residual thresholds range from $1\;\times \;10^{-4}$ to $1\;\times \;10^{-3}\;V^{2}\;m^{-2}$; see [21] for further details on the precision of BEM-FMM utilized.

**Adaptive mesh refinement:** Adaptive mesh refinement (AMR) is a general technique that ensures the convergence of the discretized solution to the BEM problem to the true analytic solution [70]. The AMR method in the context of BEM-FMM was introduced in [22] (also see [21]).

AMR consists of subdividing mesh triangles according to a cost function until a stopping criterion, based on the convergence of the solution over a region of interest, is reached. In our case, the cost function for the *m*-th triangular facet is $C_{m}=\left|\rho_{m}\right|\;\cdot \;A_{m}$, where $\rho_{m}$ is the charge density in the facet, and $A_{m}$ is its area. In other words, our cost function is the total charge magnitude in each triangle. The triangles with the top 1% cost over all triangles are subdivided into four congruent triangles. After this, the surface charges are recomputed using BEM-FMM, new costs are computed, and a new AMR step begins. The AMR process is terminated whenever the change in electrode voltages is less than 1% relative to the previous AMR step; to be precise, let $V_{n}\in R^{256}$ be the measured electrode potentials at the *n*-th AMR step. We stop the iterative procedure at the *n*-th step provided that

(7) $$∥V_{n}-V_{n-1}{∥}_{2}/{∥V_{n-1}∥}_{2}<0.01,$$

where ${∥\;\cdot \;∥}_{2}$ is the 2-norm in $R^{256}$. This stopping criterion has a simple interpretation: Once the relative change in voltage values is very small (less than 1%), we consider that the AMR algorithm has converged to a good approximation of the analytical solution.

When we perform AMR, all layers of the model are refined except for the skin layer, which is our region of interest, and it is used to compare the relative change from one AMR step to the next.

The AMR implementation that we used is the one utilized and described in [21]. In [71], the reader can find error analyses of BEM-FMM with AMR against the analytical solution of the concentric spheres model for EEG. We did not perform particular error estimates against the true solution of the EEG forward model in the five-layer model. We mention, however, that the AMR solution is guaranteed to converge to the analytical solution under certain assumptions [70].

**Figure 6:**
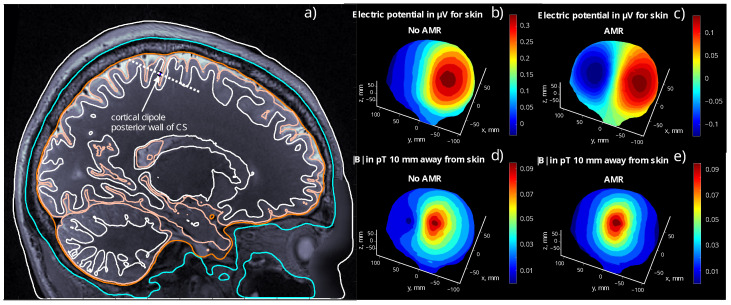
A comparison between the five-layer EEG and MEG forward solutions for dip1 and subject 110411 using the SimNIBS7 conductivity set with and without AMR. In Figure (**a**), we see the sagittal view of the subject. The colors of the lines correspond to the following layers: white (outer), skin; teal, skull; orange, CSF; apricot, GM; and white (inner) WM. In Figures (**b**,**c**), the colors indicate the value of the potential differences in μV with respect to a zeroth electrode placed on the forehead of the subject. In Figures (**d**,**e**), the values represent the strength of the magnetic flux in pT,10mm away from the subject’s skin. Notice the vast differences in the EEG solution profiles: without AMR (**b**), the forward solution does not exhibit the expected skin voltage profile of a tangential dipole; instead, it shows a profile that would be expected from a radial dipole. However, when AMR is used (**c**), we observe the typical profile pattern of a tangential dipole. In this particular instance, the source location error with the three-layer BEM for the potentials in (**c**) is 5.6mm with an orientation error of $13^{\circ }$, and the location error for the potentials in (**b**) is 33mm with an orientation error of $56^{\circ }$. Discrepancies in the profile are also observable in the MEG forward solutions (**d**,**e**), although somewhat less pronounced.

AMR has been incorporated to ensure the accuracy of the five-layer forward solution since the proximity of the dipole source singularity to the triangular elements of the mesh can cause large numerical errors; see Figure 6. AMR has never been applied to the dipole fitting with three-layer models, as this is not part of Fieldtrip’s three-layer bemcp BEM implementation.

**EEG source localization:** For EEG source localization, we used the FieldTrip MATLAB Toolbox [14]. We used the functions ft_prepare_headmodel with the three-layer decimated meshes, using each of the conductivity values in Table 2. A single-dipole source localization (with free dipole position and orientation) was carried out using nonlinear optimization [72] via the FieldTrip function ft_dipolefitting. The forward solver used in the dipole-fitting procedure was bemcp [73], which implements the classical surface potential formulation of the EEG forward problem [17,74] using a three-layer BEM model. We note that this forward solution does not include AMR.

For each subject and dipole position, the input data for the source localization were the data generated from the five-layer BEM-FMM models, using each of the five-layer conductivity sets: IT’IS7, SimNIBS7, and VWB7. The subsequent dipole localization was carried out using the corresponding three-layer conductivities: IT’IS3, SimNIBS3, and VWB3.

The “best fit” for the dipole was reported according to a least-squares approximation of the ground truth EEG data by the EEG data generated using the fitted dipole. The accuracy of the dipole fit is, thus, measured in terms of residual variance (RV), which is the variance in EEG data unexplained by the dipole fit. Therefore, smaller values of residual variance indicate a better fit.

We carried out two dipole-fitting strategies, and in every case, we reported the predicted dipole that has the smallest residual variance. In the first method, we start a nonlinear fit with the true source dipole position as an initial value for the inverse problem [72]. In the second method, we prepare a grid with a resolution of 5 using the function ft_prepare_sourcemodel, and a grid search is performed from every grid position within the skull. The best initial position in the grid is then used as the initial point of a nonlinear fit.

## 3. Localization Error Maps on the Grey Matter Surface of Each Subject

**Outline of the procedure:** For each subject, we placed 4000 point dipoles along the midsurface between grey and white matter shells and computed the corresponding forward solution using BEM-FMM. These 4000 forward solutions were computed with five-layer Headreco segmentation models and conductivity values given by the SimNIBS7 set; Table 1. Due to the large number of dipoles, we used a refinement heuristic called “*b*-refinement”, which was recently introduced in [71] and dramatically reduces the number of iterations required to obtain an accurate forward EEG solution.

The dipole fitting is carried out using the three-layer BEM model with SimNIBS3 conductivities, as in Section 2, with the only difference being that this time, we do not perform the computationally expensive grid search. We observed that in most cases, the distance between the fitted dipoles in the grid search and in the search starting at the source point is of the order of 10 μm or less. Therefore, the error calculations should not differ considerably between both methods.

**Figure 7:**
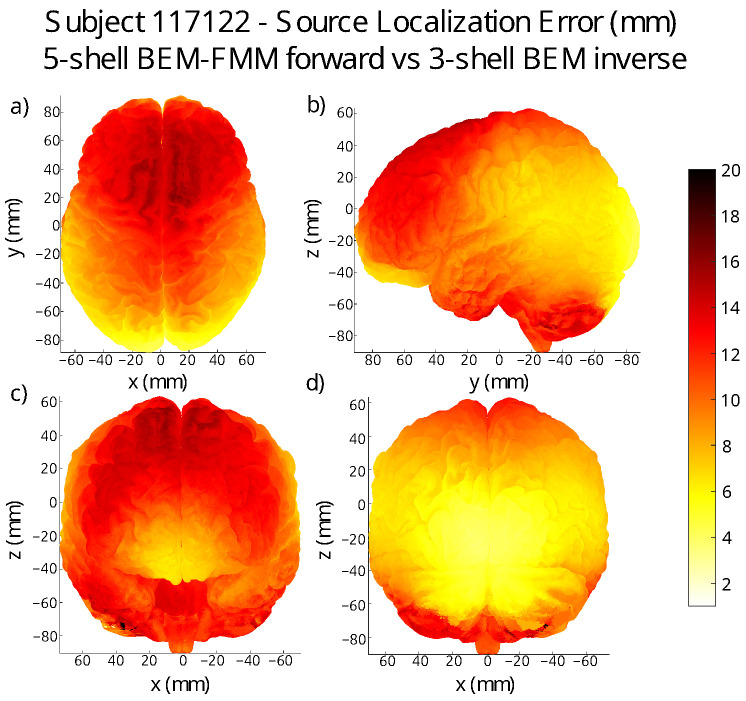
Error map for subject 117122: the lighter color corresponds to a better dipole fit location accuracy when using the three-layer BEM with five-layer BEM-FMM+*b*-AMR as the forward method. (**a**) transverse view; (**b**) left sagittal view; (**c**) anterior coronal view; (**d**) posterior coronal view.

**Figure 8:**
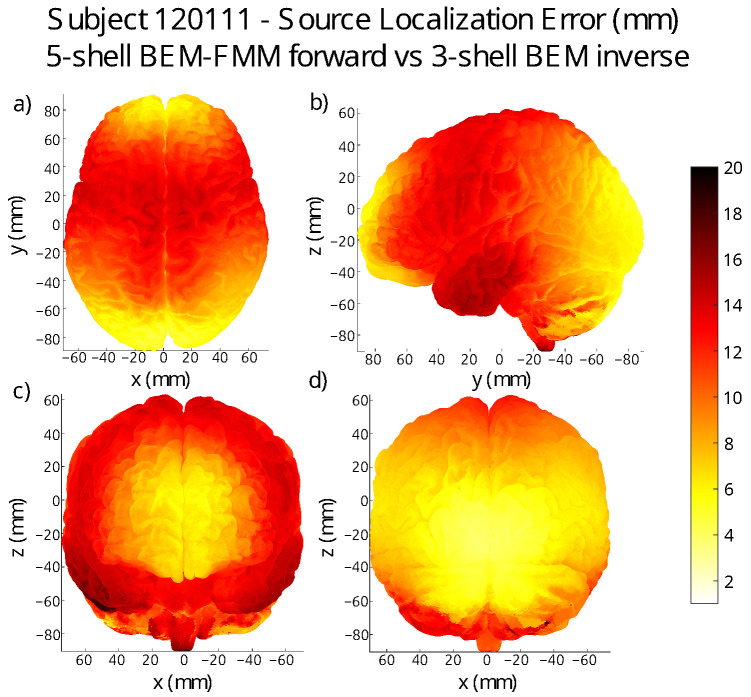
Error map for subject 120111. (**a**) transverse view; (**b**) left sagittal view; (**c**) anterior coronal view; (**d**) posterior coronal view.

Once the 4000 dipole fits have been calculated, we compute the distance between each fitted dipole and the dipole source. To produce an image of the distance error over the entire grey matter, we interpolate these results according to the reciprocal of the distance of each triangle center to each dipole source within a given interpolation radius, *R*. In other words, to a triangle with center r, we assign the error value

(8) $$\frac{\sum_{i=1}^{k}\epsilon_{i}/\left|r-s_{i}\right|}{\sum_{i=1}^{k}1/\left|r-s_{i}\right|},$$

where $\left\{s_{1},\cdots ,s_{k}\right\}$ is the set of dipole sources at a distance of ≤R from r, and $\epsilon_{i}$ is the fitting distance error at the dipole with center $s_{i}$. In Figure 7 and Figure 8, we exhibit some of the two error maps we generated.

**The method of *b*-refinement:** In conventional AMR, the cost function $C_{m}^{\left(n\right)}$ at step *n* is given by $\rho_{m}^{\left(n-1\right)}$, i.e., the surface charge density computed in the previous AMR step. In *b*-refinement (or *b*-AMR), instead of computing the full solution to the charge density in the previous step, we only compute its zero-order approximation, which is given by the incident field at the mesh triangle centers. This can be expressed as

(9) $$C_{m}=\left|E_{m}^{i}\right|\;\cdot \;A_{m},$$

where $E^{i}$ is the incident field at the *m*-th facet, and $A_{m}$ is the area of the *m*-th facet. Equation (9) does not explicitly show its dependence on the refinement step *n*, but it varies with it since the meshes are subdivided according to the previous computation of $C_{m}$. In [71], the authors study the accuracy of *b*-refinement using a concentric spheres model, where the analytic solution is known, as well as a high-resolution five-layer head model using BEM-FMM with conventional AMR as a reference solution. Their findings show that in the high-resolution headmodel, *b*-refinement exhibits an RDM error of under 2% and a 2-norm error of under 5% for the EEG forward solution.

## 4. Results

**Summary of results:** The average distance error in source localization for our four selected locations (across all subjects, segmentations, and conductivities) caused by the discrepancy in charge-based BEM-FMM and potential-based BEM is ∼1mm in a three-layer model. The difference in the forward methods, together with the discrepancy between the three- and five-layer models, causes an average distance error of ∼4.5mm.

The average error over the entire grey matter surface —using the SimNIBS7 conductivity set, and the Headreco segmentation—is ∼9mm across all subjects. The patterns of regions of high- and low-dipole-fitting quality appear to be unique to every subject. The main trend that we observed is that the quality of dipole fit tends to be better in the lateral and posterior regions of the brain for most of the subjects, although there are clear exceptions to this.

**Three-layer model comparison:** FieldTrip does not use the charge-based BEM-FMM in its forward engine; instead, it uses the classical potential-based formulation of the BEM [17,74]. Because of this, we made a model comparison to estimate the baseline accuracy of FieldTrip’s potential-based three-layer inverse model (which does not use AMR) under the assumption that the forward charge-based BEM-FMM+AMR solution calculated using the three-layer model is ground truth. This is a necessary step since there is no analytic solution available, and by knowing the discrepancy due to model differences, we will be able to better assess the decrease in the accuracy of dipole fitting due to the inclusion of additional layers.

First, we calculated the potentials in the entire SKIN layer using a forward three-layer model with charge-based BEM-FMM and AMR. Then, we retrieved readings for the electrode potentials from this calculation using the potential value at the SKIN triangle corresponding to each electrode position. With these electrode voltages, we generated a raw EEG dataset (compatible with FieldTrip software) consisting of a single time sample. Finally, we performed EEG source reconstruction from the simulated EEG using a FieldTrip three-layer volume conduction model with the same conductivity values used in the forward model. See Figure S17 for a diagrammatic representation of the three-layer model comparison process.

The FieldTrip inverse model retrieves the source dipole position from three-shell BEM-FMM+AMR data with an average distance error of ∼1mm (over all dipoles, subjects, and models) and an average angle error of $0.75^{\circ }$. Detailed tables with three-layer model comparison values can be found in Table S13. Comparison tables without the use of AMR for the input data can be found in Table S14.

**Five-layer vs. three-layer results:** We generated the forward BEM-FMM+AMR solution from each dipole location and each five-layer model, assuming that these solutions are ground truth. Then, we performed dipole fitting using the three-layer model of the corresponding conductivity as an inverse model (e.g., when using IT’IS7 as the conductivity set for our forward computations, we used IT’IS3 as the conductivity set for the inverse computation); see Figure S16 for a flowchart figure. We measured the distance error and orientation error in each case.

We obtained an average distance error (across all dipole positions and subjects) of ∼4mm (∼1mm std.) using FreeSurfer meshes and an average distance error of ∼4mm (∼2.5mm std.) using Headreco meshes. The error in dipole orientation is more pronounced, with ∼$15^{\circ }$ average (∼$10^{\circ }$ std.) for FreeSurfer models and ∼$20^{\circ }$ average (∼$20^{\circ }$ std.) for Headreco models. See Table 3 for dipole-wise results.

For additional tables containing data related to the 3-layer model comparison see Tables S5, S6, S13, and S14. For model-wise results for three-layer vs. five-layer models with and without AMR, see Tables S9–S12.

**Subject error maps:** We located 4000 dipoles in the midsurface between the grey and white matter of every subject. This placement was carried out by first creating a *k*-means clustering of the grey matter shell and then placing dipoles in the closest midsurface point to each cluster center. We generated the forward solution for each of the 4000 dipoles in isolation using BEM-FMM coupled with *b*-AMR, and we performed dipole fitting on each of the corresponding voltage readings. This produced a data vector of 4000 distance error values corresponding to each of the dipole centers. Once we had this data, we interpolated it to obtain error values for all the triangle centers of the grey matter mesh; see Figure 7 and Figure 8. The complete set of errormap figures can be found in Figures S1–S15.

Table 4 summarizes the basic statistics associated with each of the error maps that we produced. Subject 122317 is an outlier of our sample; for this subject, the left hemisphere presents much larger localization errors than the right hemisphere. All other patients present largely symmetrical and smooth error maps, and most of them exhibit better accuracy in the posterior and lateral areas of the grey matter.

## 5. Discussion

**Selected dipole fittings:** Among the four selected dipoles considered, dip4 has the worst localization results, which is perhaps to be expected since its location in the medio-temporal region (Figure 4) is the deepest of the four. More surprisingly, the position of dip2 (a radial dipole located in the ${M1}_{\mathrm{HAND}}$ region) was the one that gave the second-to-worst results. The reason may be that the three-layer models do not include CSF, and the closer proximity of the dipole to the highly conductive CSF layer creates a larger discrepancy between the five- and three-layer models. However, one should not over-interpret these results, as more tests for radial dipoles with varying thicknesses of neighboring CSF layers may be needed before a conclusion can be made.

**Figure 9:**
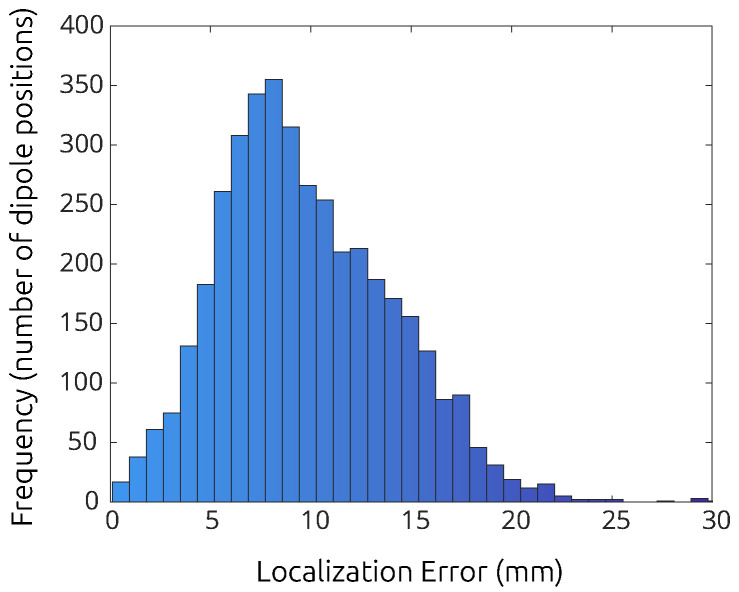
Histogram of dipole-fitting error (mm) over 4000 well-separated dipole locations across the grey matter surface of subject 117122.

**Advantages and shortcomings of the three-layer model:** The residual variances we obtained (circa 0.0015 on average) are extremely small, which indicates that the nonlinear dipole fitting using the three-layer model explains most of the EEG data variance with the predicted positions. This indicates that these are good-quality fits. The three-layer model retrieves dipole locations and orientations with good average accuracy from noiseless data. However, when inspecting the histogram of the dipole-fitting errors, one can see common occurrences of errors above 2 cm (Figure 9). Given that we used AMR and *b*-AMR for our experiments, these errors can be attributed mostly to the discrepancy between model complexity. This suggests that more complex models of five or more layers may be necessary for accurate source reconstruction.

The reader should be aware that, although we did not attempt to model noise in this study, the dipole-fitting errors may be significantly larger when the data is noisy, as is the case with real EEG data. Despite its shortcomings, the three-layer BEM model remains a useful tool for source localization, as it is very computationally efficient and does not typically require refinement methods.

**Figure 10:**
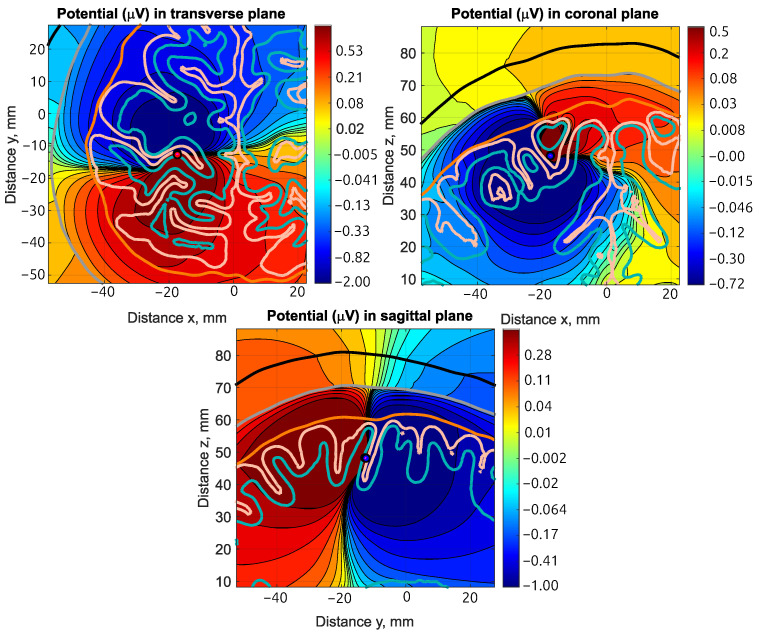
Volumetric plots using a logarithmic scale of the electric potential (μV) around the source dipole dip1 (left hemisphere, posterior wall of the central sulcus opposite to M1 hand region) in subject 110411 for transverse (top anterior; bottom posterior), coronal (top superior; bottom inferior), and sagittal (left posterior; right anterior) views. The colors indicate the varying values of the potential, with the red shades indicating positive values and the blue shades negative values. In the absence of any tissues, the volumetric plots would exhibit mirror symmetry across the plane passing through the dipole center and perpendicular to the dipole direction. The presence of the white matter (highlighted in teal) and the grey matter (highlighted in apricot color) produce a symmetry break that would not occur in a three-layer model; this is particularly noticeable in the sagittal view. Clearly, the symmetry break will contribute to errors in the determination of dipole orientation.

**Advantages and challenges of the five-layer model:** Inclusion of the grey and white matter tissues is a major factor in the variation of dipole orientation (see Figure 10); this is in agreement with the findings in [10].

Model-wise, the conductivity set that produces the best agreement between the three-layer and five-layer models is IT’IS. A possible explanation is that this set has the lowest conductivity contrast between grey matter and CSF. We also observe (comparing Tables S9 and S10 against Tables S11 and S12) that the dipole fits for the forward data generated with Headreco models have higher variability than those using the data from the FreeSurfer models (both in terms of location and orientation). The use of AMR theoretically eliminates the discrepancies caused by the different number of triangles in the initial layers; therefore, a possible explanation for the different variabilities is that Headreco meshes use a more realistic skull layer than the FreeSurfer meshes. This explanation aligns with the results in [11]. However, a formal assessment of the effect of mesh uncertainty would require a more detailed analysis using techniques similar to those in [10,44].

Figure 6 shows that it is necessary to incorporate some form of mesh refinement to have accurate forward computations and, in turn, an accurate dipole fitting based on five-layer BEM models. Certain dipole positions require up to 12 conventional AMR steps before they can reduce the electrode error sufficiently. For this reason, a practical implementation of dipole fitting using BEM-FMM would require the use of *b*-AMR or some other accurate heuristic. This is in contrast to three-layer models: the lack of intracortical compartments implies that the dipolar sources will be typically well-separated from the model meshes, and thus, the error caused by the lack of AMR is much lower.

**Implications for practical source reconstruction:** The error maps in Figure 7 and Figure 8 suggest that single-dipole EEG source localization may be improved for sources located on the frontal and parietal lobes with the use of higher-resolution models, as these are the areas where the three-layer BEM model typically performs worse. However, this is not a general rule, as for certain subjects, the three-layer BEM performs worse on the occipital lobe; see for example Figures S1 and S9.

The method of *b*-AMR with BEM-FMM appears to be feasible for practical source reconstruction with five-layer models, given our results and the time-performance and error analysis results presented in [71]. Given that our proposed method is also applicable to three- and four-layer models, it would be interesting to analyze the possible performance improvements with high-resolution versions of these mesh models. A future direction to better assess the source localization improvements of high-resolution models would be an analysis using phantoms [12] or experimental subject data [75].

## Figures and Tables

**Table 1:** Conductivity values (${S\;m}^{-1}\;$) used in the five-layer/seven-compartment models.

	VWB7	IT’IS7	SimNIBS7
Skin	0.430	0.147	0.465
Skull	0.010	0.0179	0.010
CSF	1.790	1.880	1.654
GM	0.330	0.419	0.275
WM	0.140	0.348	0.126
Cerebellum	0.216	0.577	0.126
Ventricles	1.790	1.880	1.654
Eyes	1.790	1.880	1.654

**Table 2:** Conductivity values (${S\;m}^{-1}\;$) for the three-layer models. The values for SKIN and SKULL are taken to be the same as in the corresponding tissues of Table 1. The conductivity of the combined BRAIN layer in the three-layer model has been taken to be 0.33 ${S\;m}^{-1}$ according to [45], except for IT’IS3, where their own value was provided in the dataset.

	VWB3	IT’IS3	SimNIBS3
SKIN	0.430	0.147	0.465
SKULL	0.010	0.0179	0.010
BRAIN	0.330	0.375	0.330

**Table 3:** Dipole- fitting errors per dipole position over all five-layer models using a BEM-FMM + AMR forward solver for the input voltages and a three-layer classical BEM solver for dipole fitting. The label RV denotes the residual variance of the fit, and AMR denotes the total number of AMR steps required for the convergence of the forward solution.

**Dipole**	**mm**	**deg**	**RV**	**AMR**
dip1	4.13	8.71	$6.88\;\times \;10^{-4}$	6.22
dip2	5.01	14.62	$8.64\;\times \;10^{-4}$	5.66
dip3	2.43	14.04	$9.87\;\times \;10^{-4}$	4.59
dip4	6.42	11.00	$8.39\;\times \;10^{-4}$	6.23
(a) Averages				
dip1	1.65	4.66	$6.39\;\times \;10^{-4}$	3.23
dip2	1.85	8.86	$5.37\;\times \;10^{-4}$	2.08
dip3	1.13	7.14	$5.69\;\times \;10^{-4}$	2.51
dip4	3.87	5.75	$5.89\;\times \;10^{-4}$	4.14
(b) Standard Deviations				

**Table 4:** Table of average dipole-fitting errors and standard deviations for 15 subjects of the Connectome Young Adult dataset. Here, dipoles located at the cerebellum and brain stem have been excluded from the computation of averages and standard deviations. Subject 122317 is an outlier in this table.

**Subject**	**Avg. Error (mm)**	**St. Dev. (mm)**
110411	10.28	3.63
117122	9.73	4.11
120111	10.11	4.48
122317	17.71	11.57
122620	5.99	2.27
124422	11.15	2.85
128632	8.05	3.47
130013	7.11	2.44
131722	9.71	3.75
138534	10.17	2.76
149337	7.41	2.51
149539	6.05	2.43
151627	5.57	2.29
160123	7.08	3.03
198451	7.41	3.07

## Data Availability

The code used to generate the results in this paper is available in the following repository: https://github.com/guillermo-nponasso/EEG-BEMFMM-5Layer.

## Supplementary Materials

The following supporting information can be downloaded at: https://www.mdpi.com/article/10.3390/bioengineering11111071/s1, Table S1: AMR—FreeSurfer; Table S2: non-AMR—FreeSurfer; Table S3: AMR—Headreco; Table S4: non-AMR—Headreco; Table S5: AMR results per dipole—3-layer model comparison; Table S6: Non-AMR Averages per dipole—3-layer model comparison; Table S7: AMR—all 7-shell models; Table S8: non-AMR results per dipole—all 7-shell models; Table S9: AMR—FreeSurfer results per model and dipole; Table S10: non-AMR—FreeSurfer results per model and dipole; Table S11: AMR—Headreco results per model and dipole; Table S12: non-AMR—Headreco results per model and dipole; Table S13: AMR—3-layer model comparison results per model and dipole; Table S14: non-AMR—3-layer model comparison results per model and dipole. Figure S1: Error map for subject 110411; Figure S2: Error map for subject 117122; Figure S3: Error map for subject 120111; Figure S4: Error map for subject 122317; Figure S5: Error map for subject 122620; Figure S6: Error map for subject 124422; Figure S7: Error map for subject 128632; Figure S8: Error map for subject 130013; Figure S9: Error map for subject 131722; Figure S10: Error map for subject 138534; Figure S11: Error map for subject 149337; Figure S12: Error map for subject 149539; Figure S13: Error map for subject 151627; Figure S14: Error map for subject 160123; Figure S15: Error map for subject 198451; Figure S16: A flowchart for the 3-layer model comparison process; Figure S17: A flowchart for the performance study of 3-layer inverse models using 5-layer forward models.
