# Supplementary material for: Improving EEG Forward Modeling Using High-Resolution Five-Layer BEM-FMM Head Models: Effect on Source Reconstruction Accuracy
Source: Bioengineering (Basel). 2024 Oct 26;11(11):1071. doi: 10.3390/bioengineering11111071 (PMC11591057; doi:10.3390/bioengineering11111071)
Supplement: Supplementary file 1 [file bioengineering-11-01071-s001.zip › figures_supplement.pdf]

Supplement 2: Figures

Below, the reader can find the computed distance error maps (mm) from 4000 dipole positions in the GM-WM interface of 15 Connectome Young Adult subjects. The forward solution was computed using a 5-layer model with BEM-FMM + b-AMR, and the inverse fit was carried with FieldTrip using a 3-layer conventional BEM model.

Followed by the error maps, we include flowcharts to the forward and inverse computations.

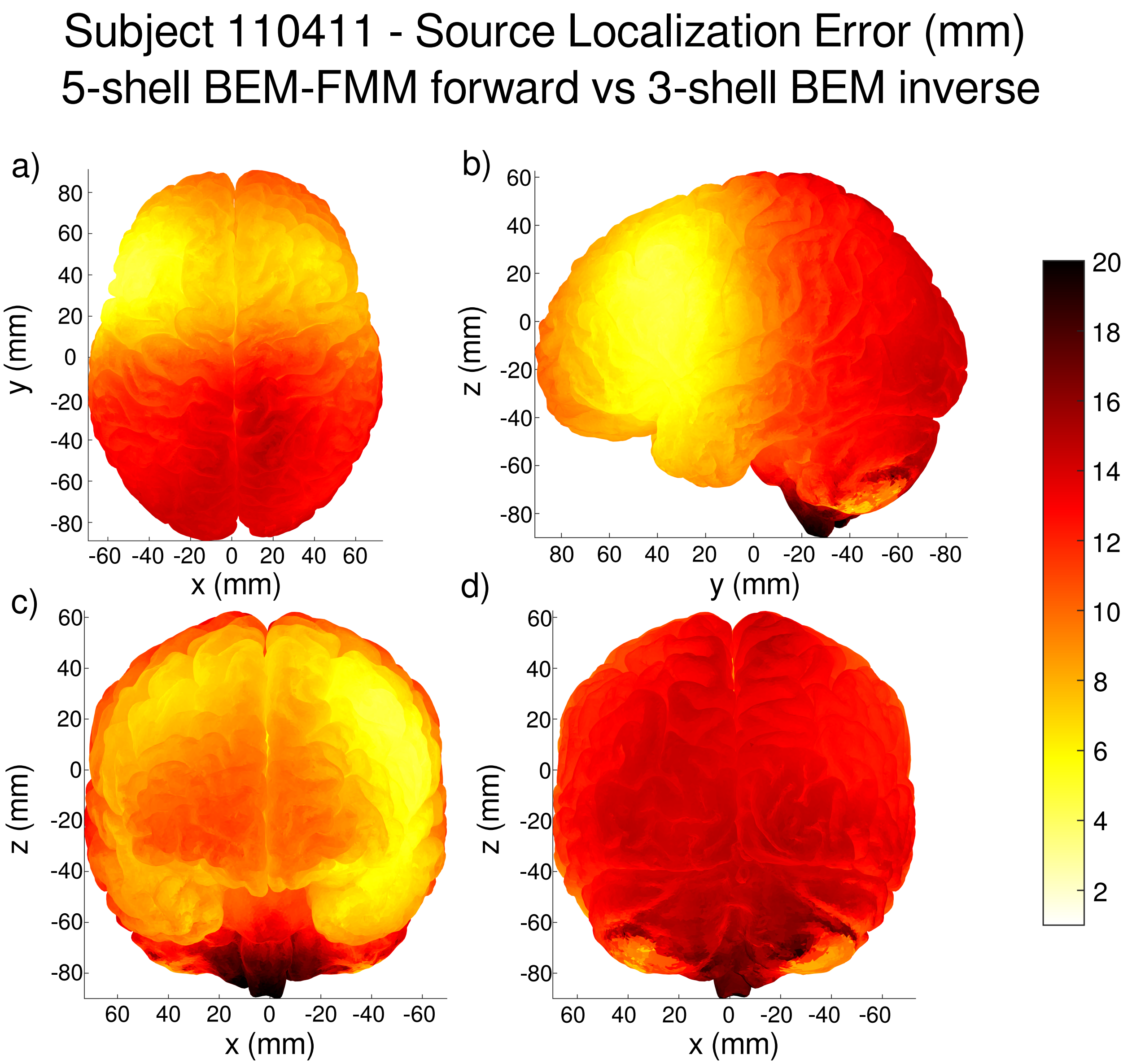

Figure S1: Error map for subject 110411.

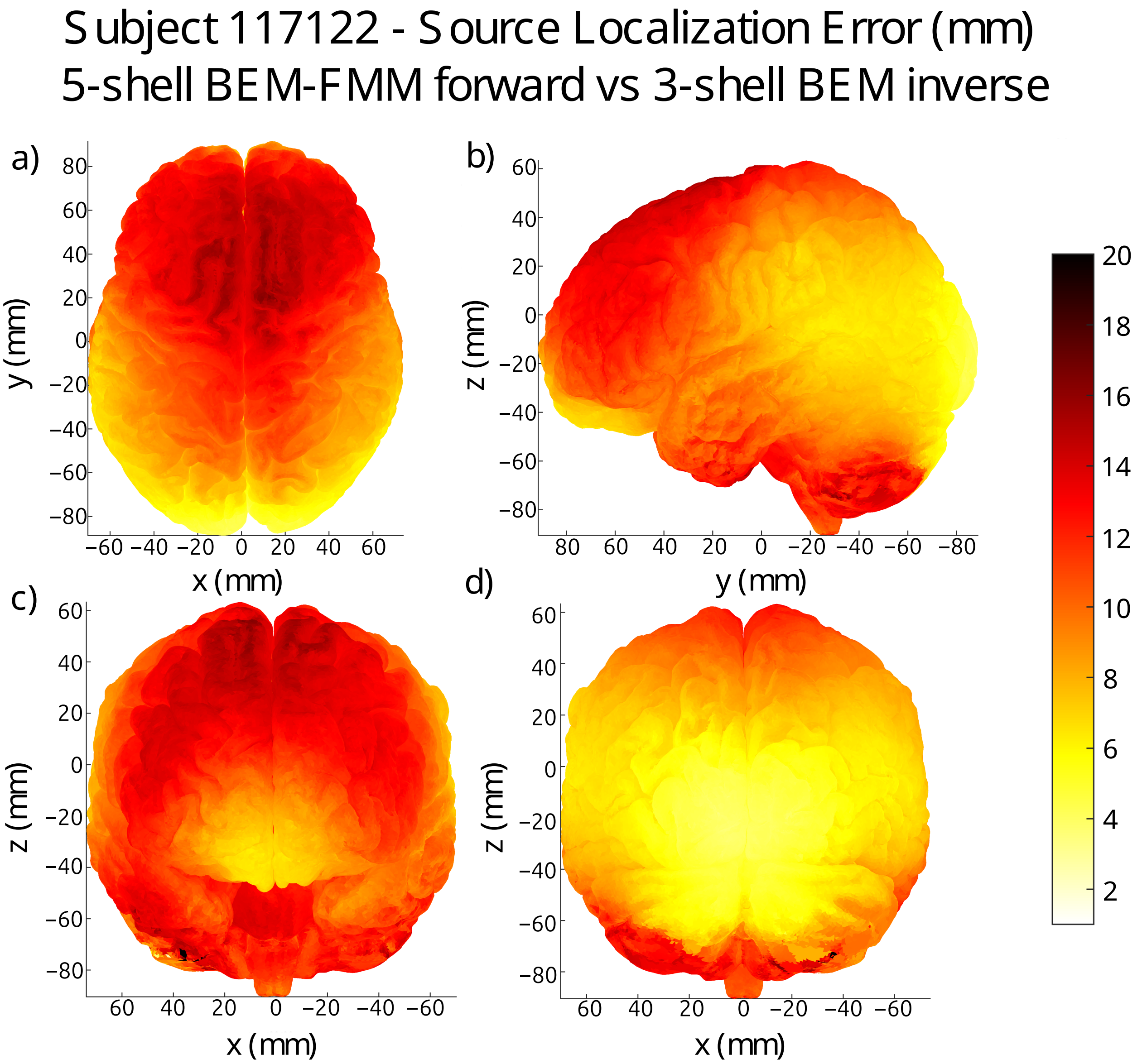

Figure S2: Error map for subject 117122.

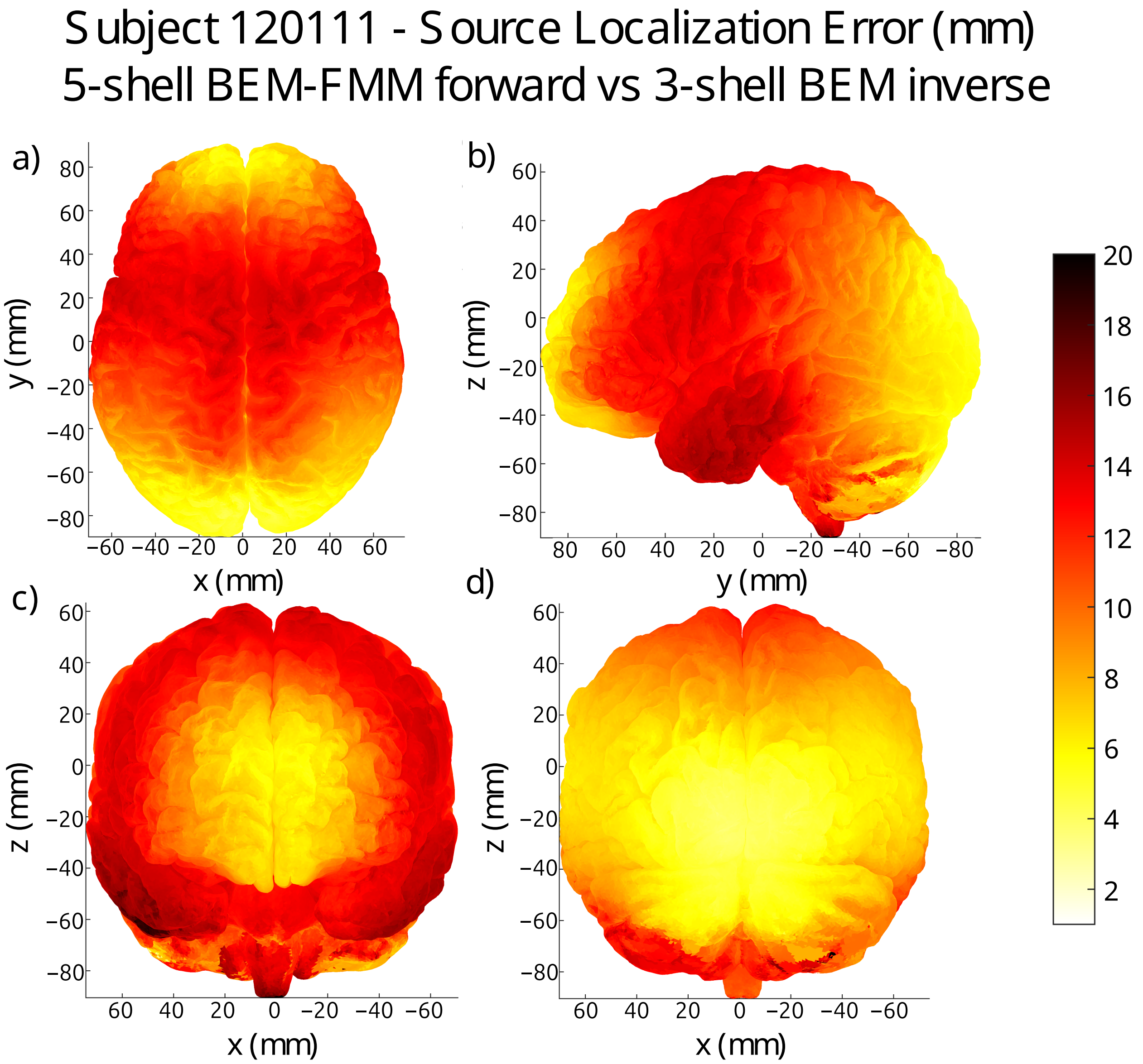

Figure S3: Error map for subject 120111.

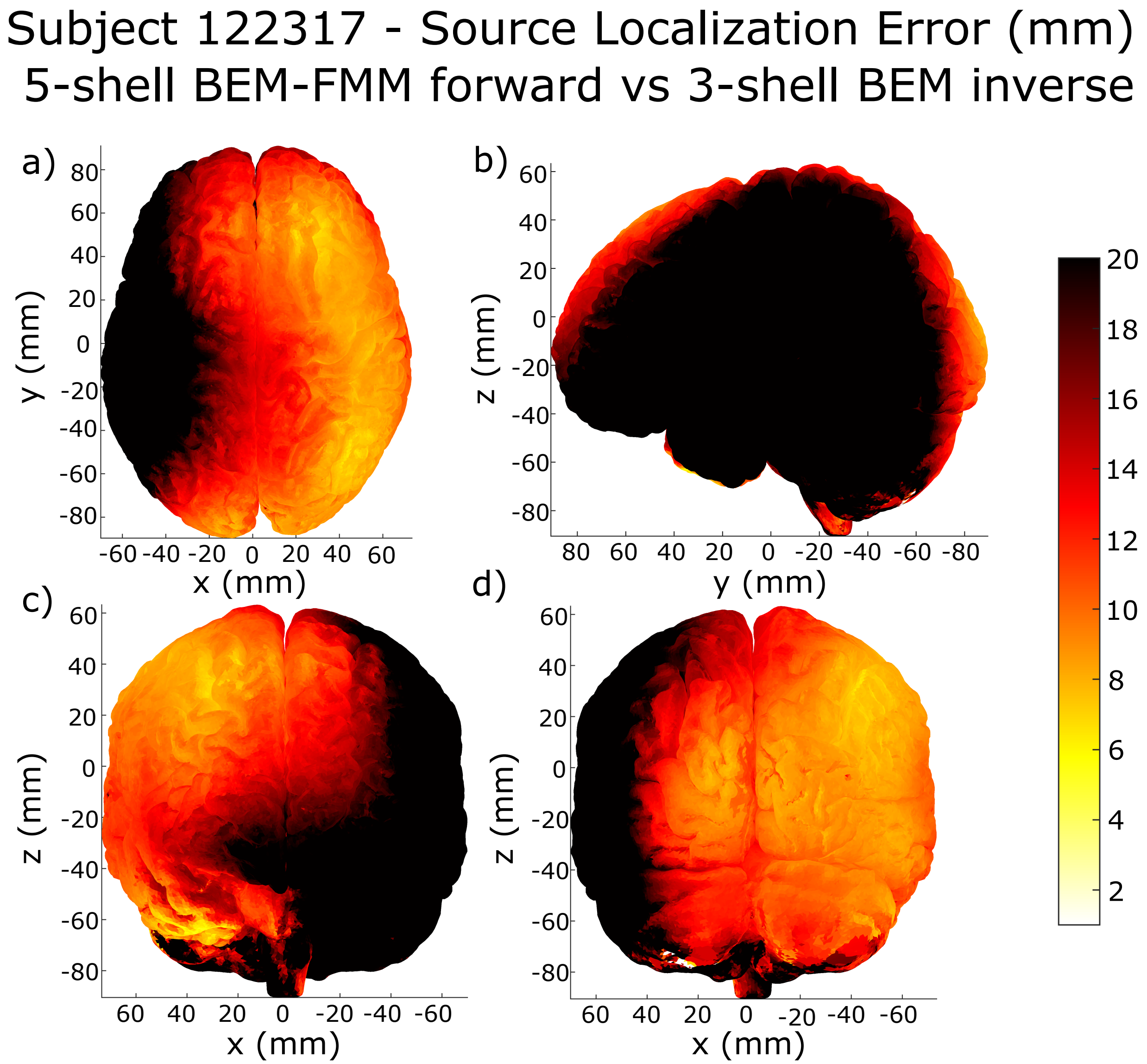

Figure S4: Error map for subject 122317.

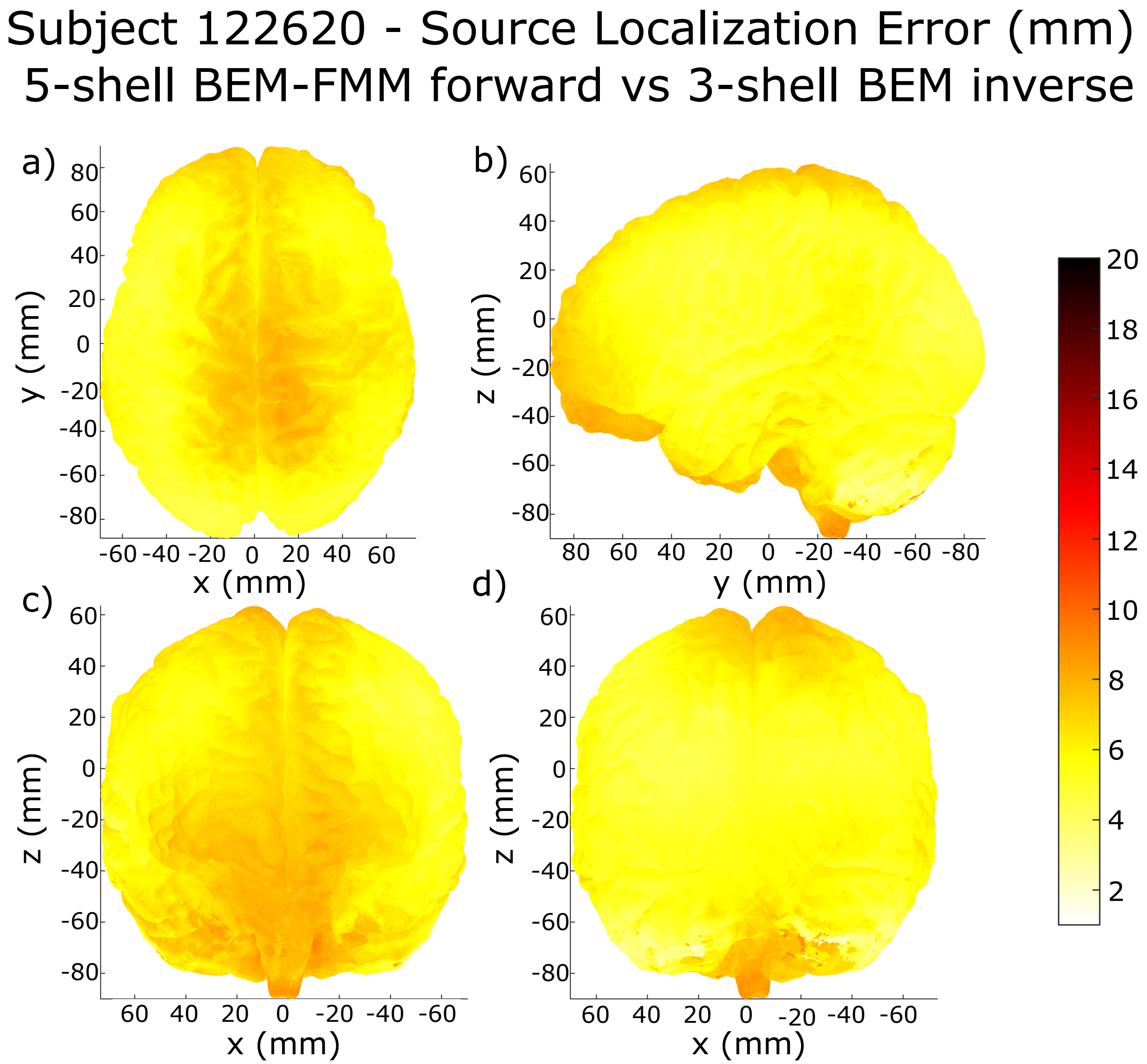

Figure S5: Error map for subject 122620.

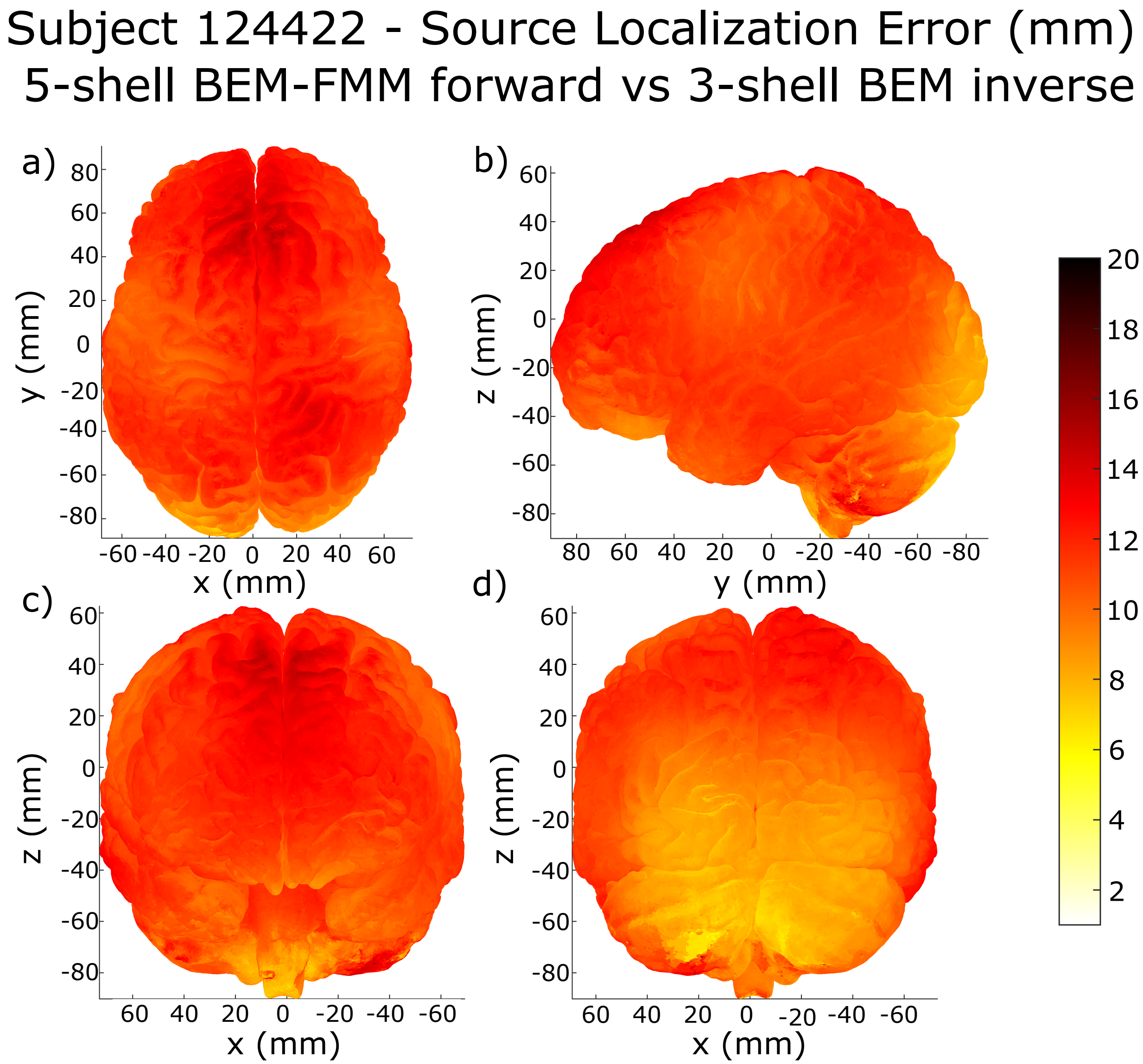

Figure S6: Error map for subject 124422.

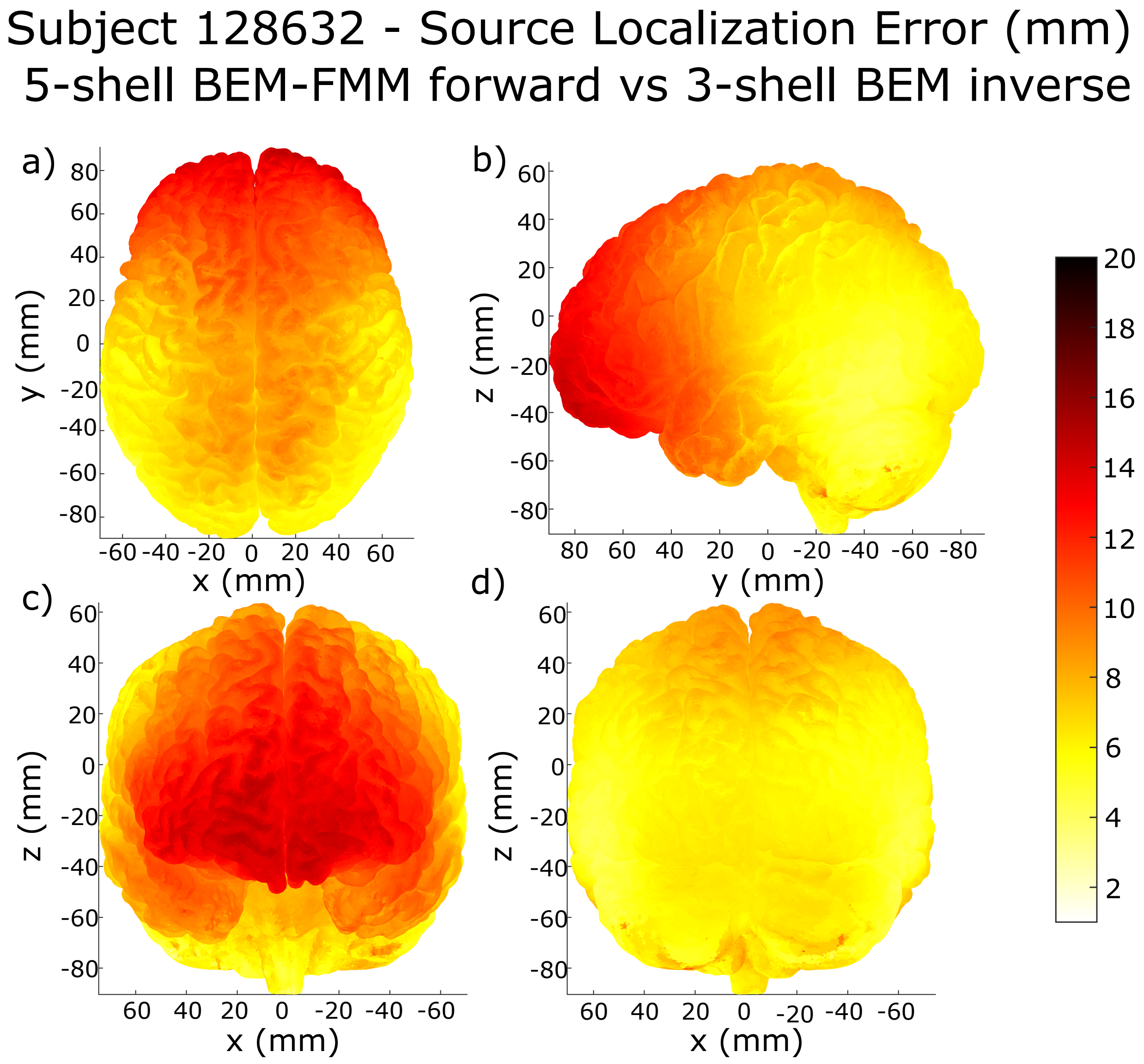

Figure S7: Error map for subject 128632.

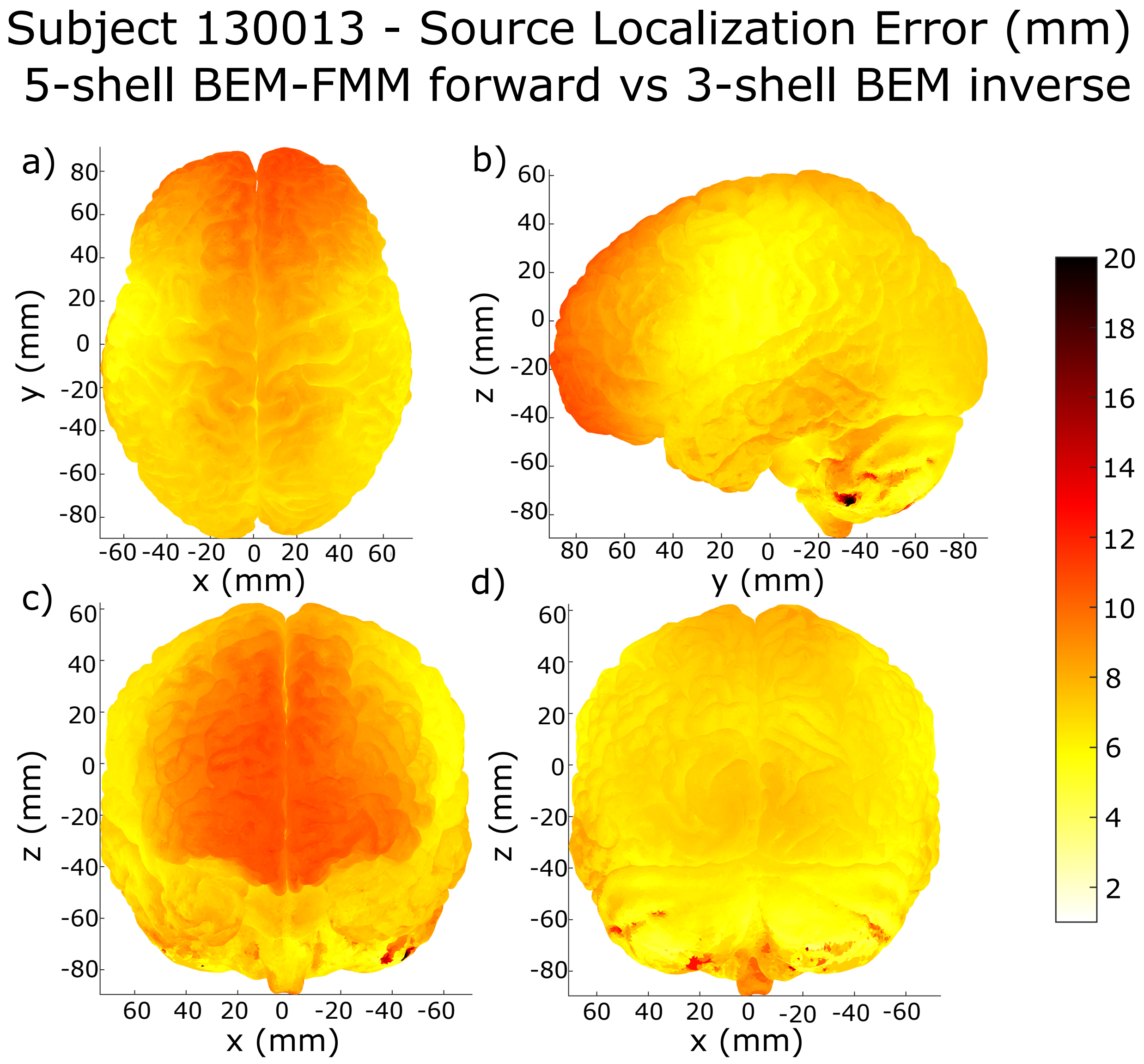

Figure S8: Error map for subject 130013.

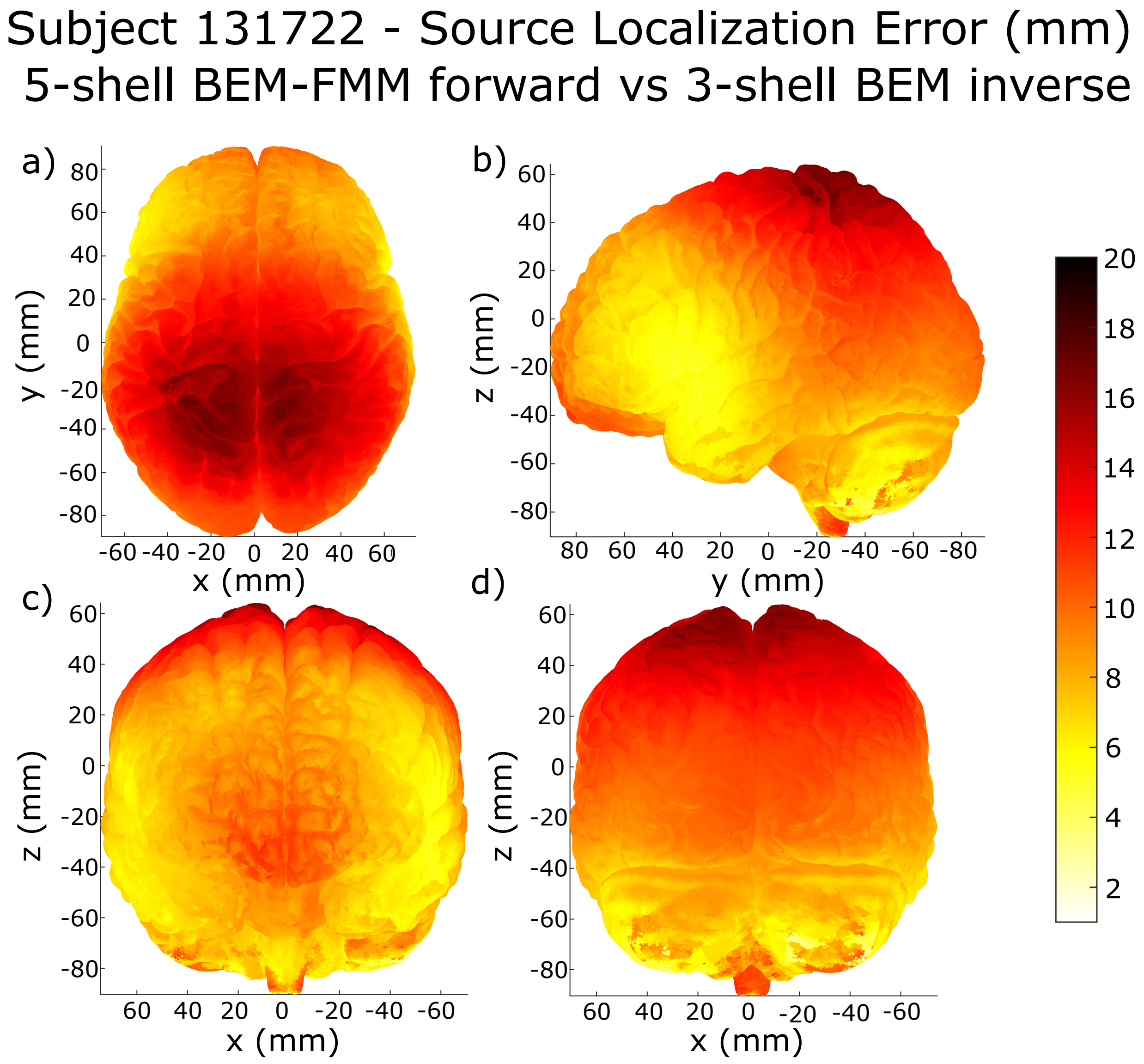

Figure S9: Error map for subject 131722.

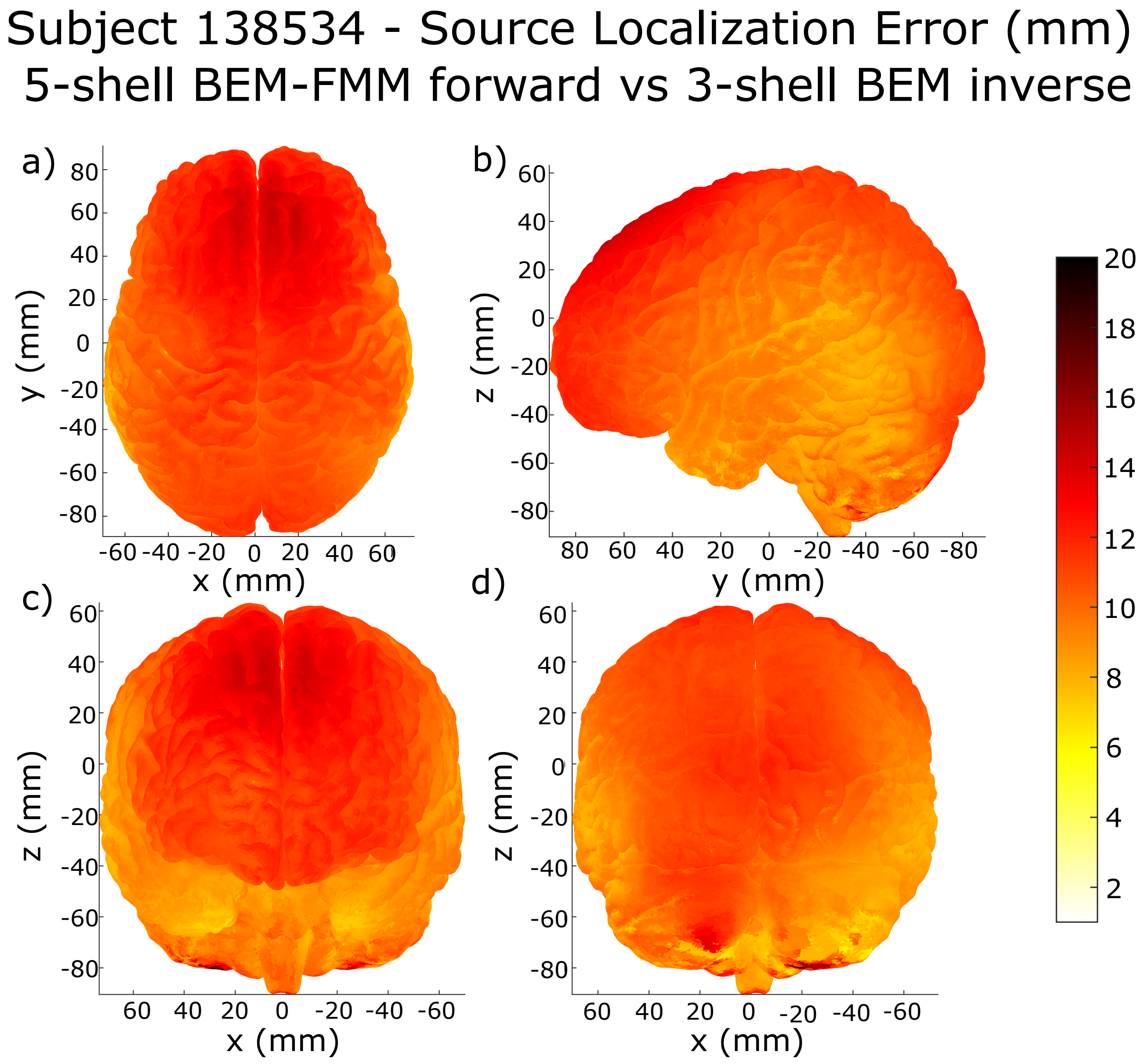

Figure S10: Error map for subject 138534.

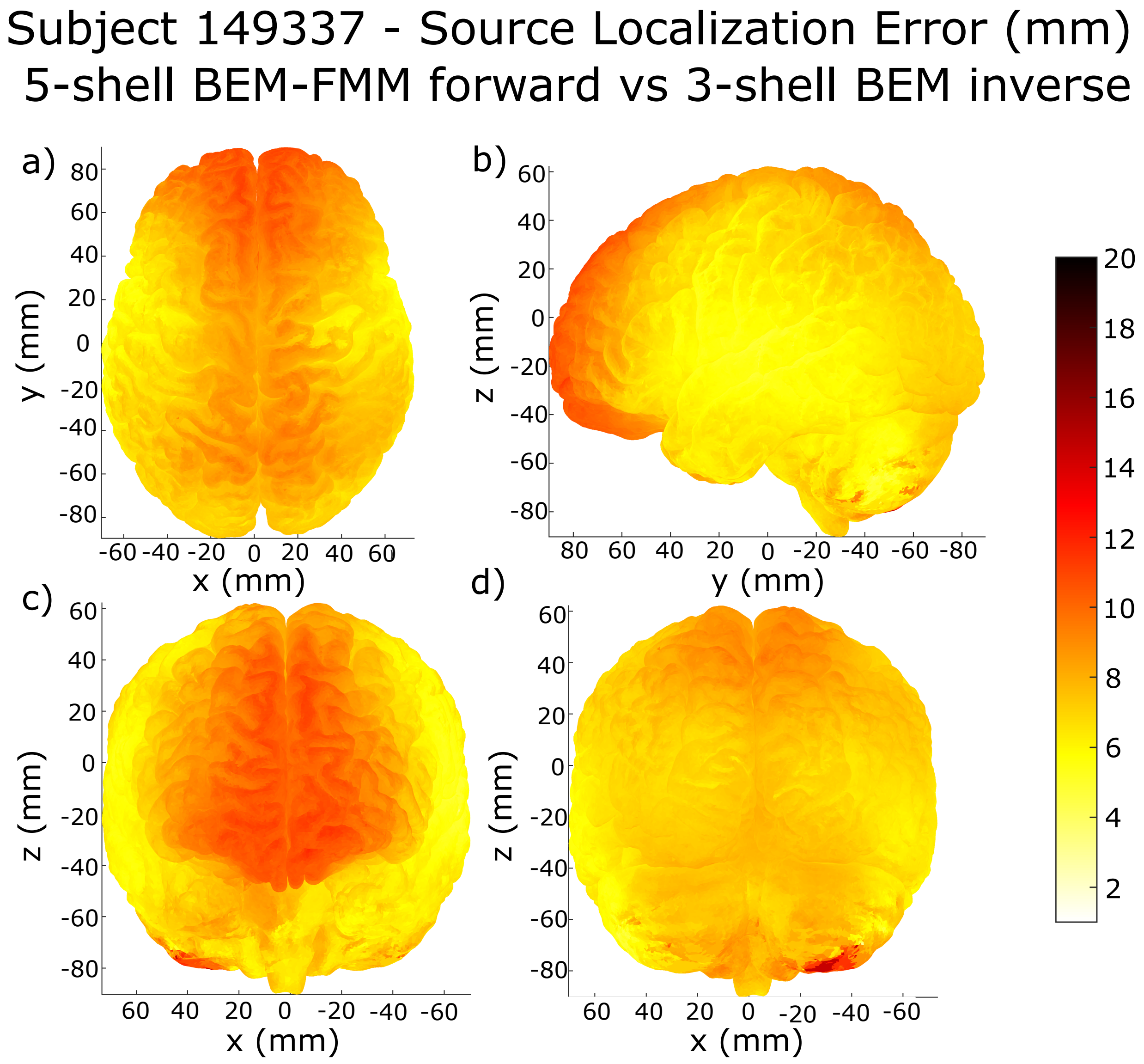

Figure S11: Error map for subject 149337.

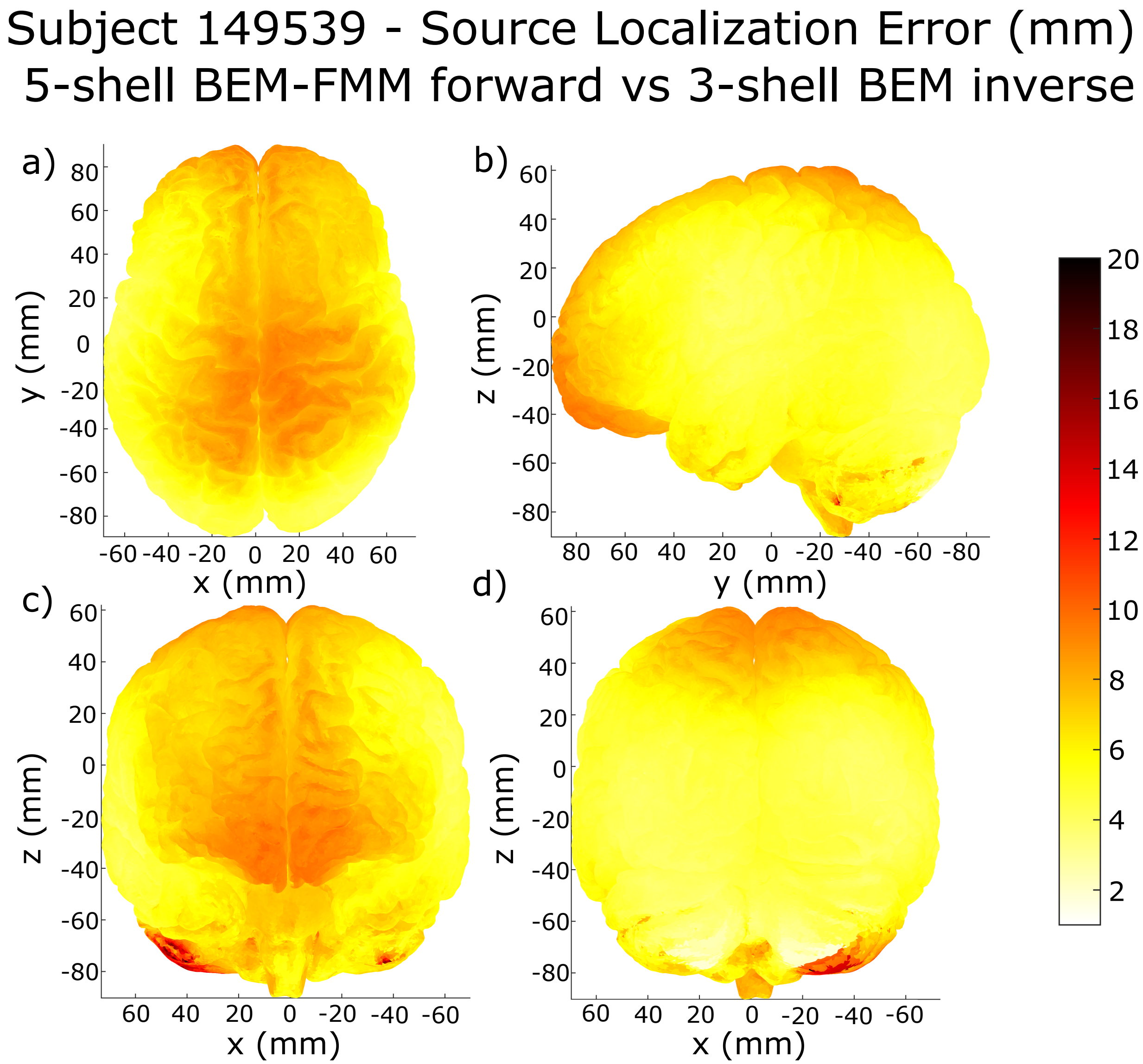

Figure S12: Error map for subject 149539.

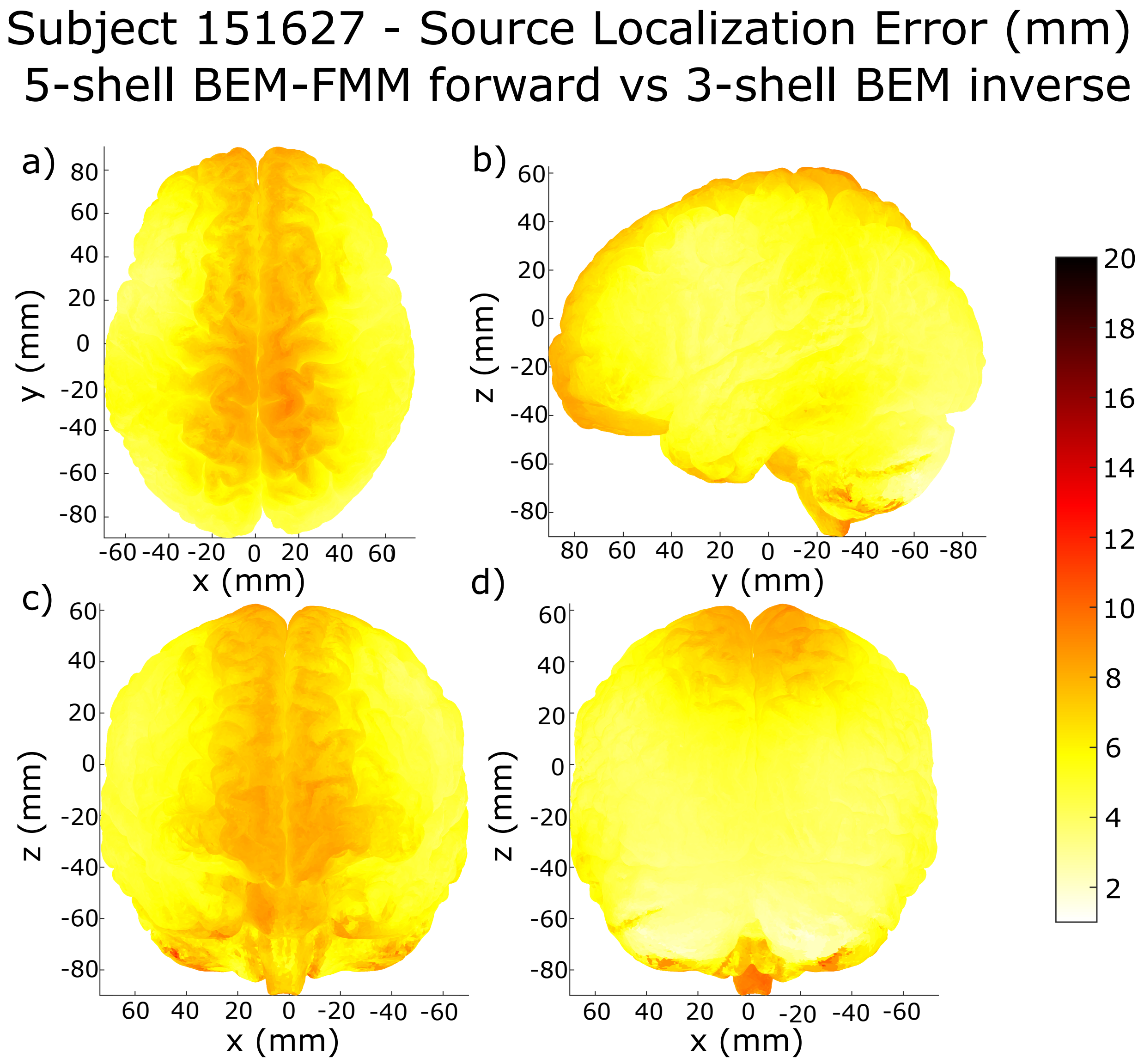

Figure S13: Error map for subject 151627.

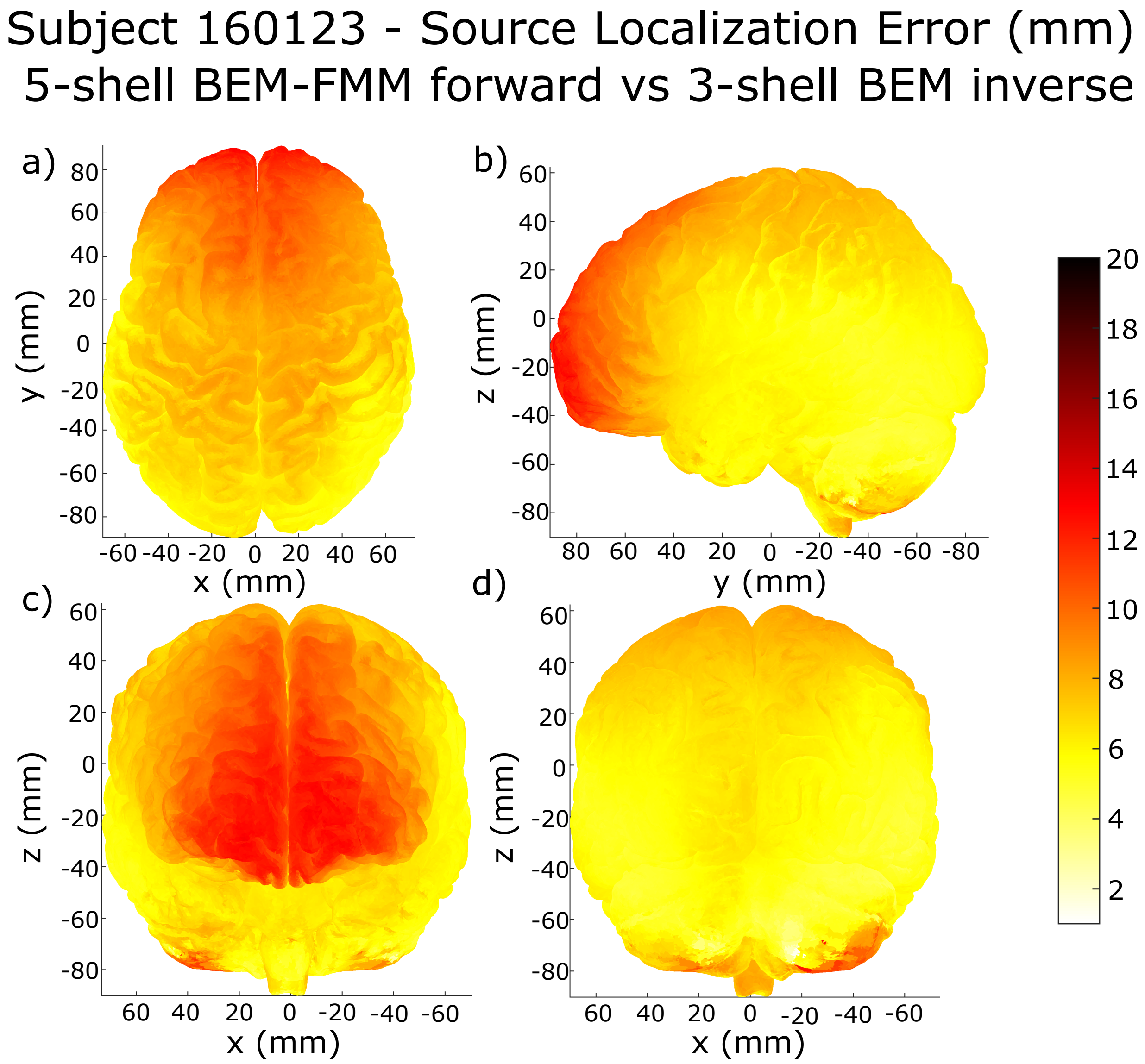

Figure S14: Error map for subject 160123.

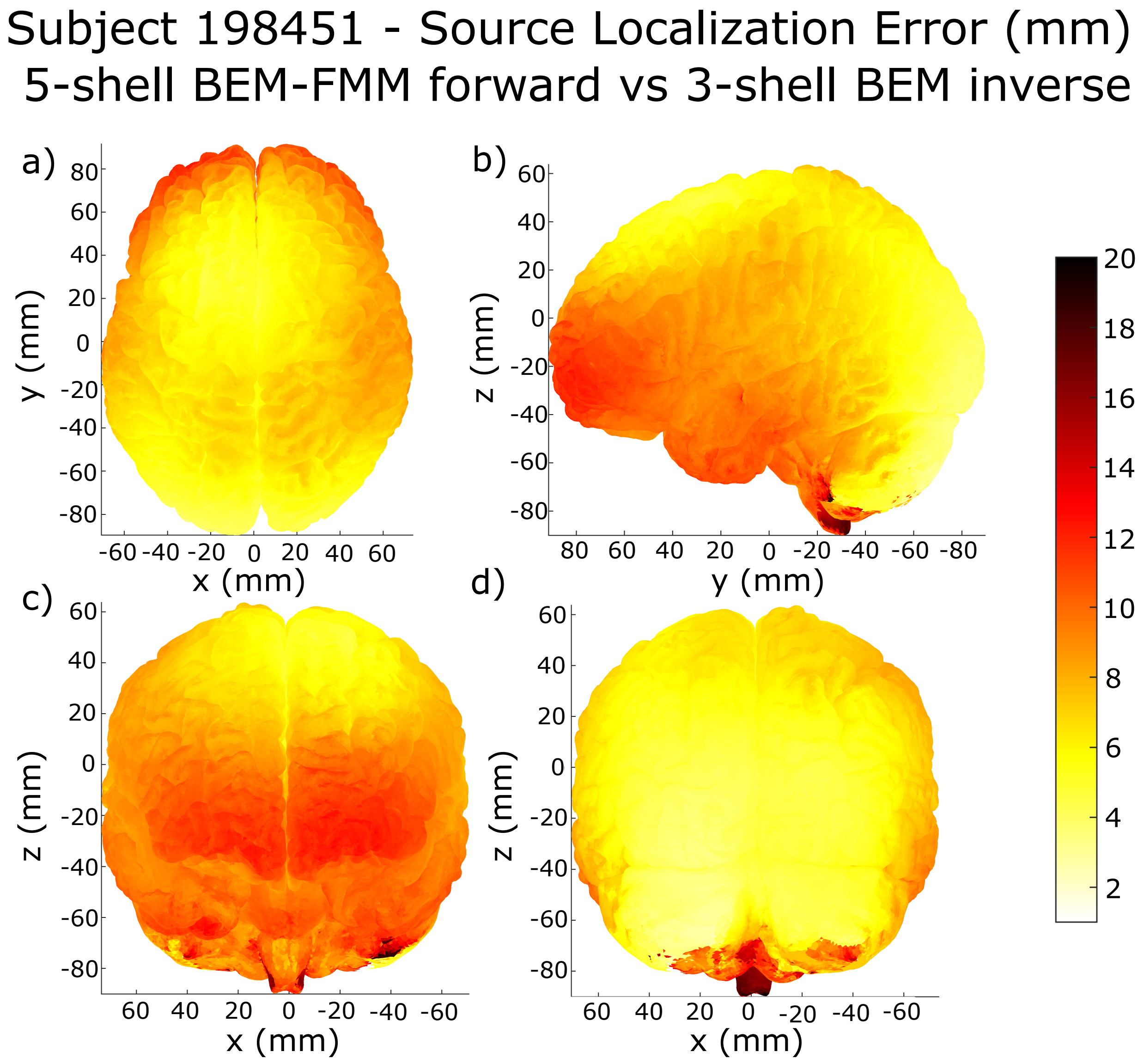

Figure S15: Error map for subject 198451.

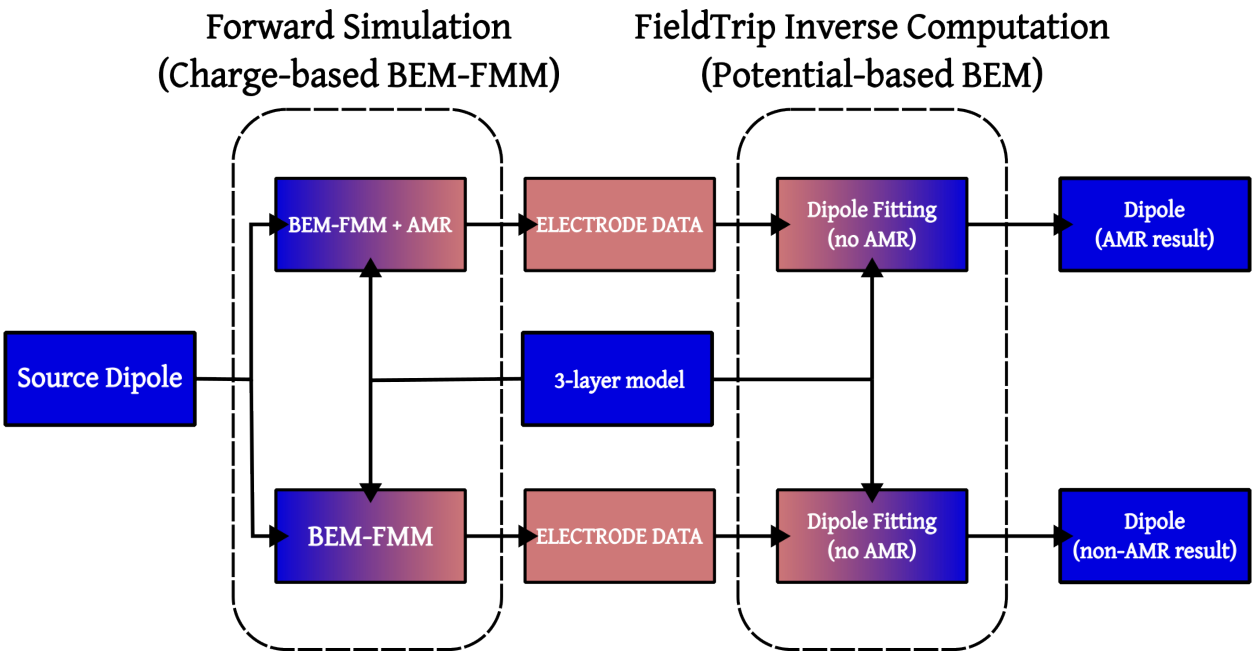

Figure S16: A flowchart for the 3-layer model comparison process. The blue shade represents geometric information, the pink shade represents electromagnetic information. Given a single source dipole and a 3-layer model (consisting of meshes and a tissue conductivity set), we produce two dipole fits: one corresponding to an AMR forward solution, and one corresponding to a non-AMR one.

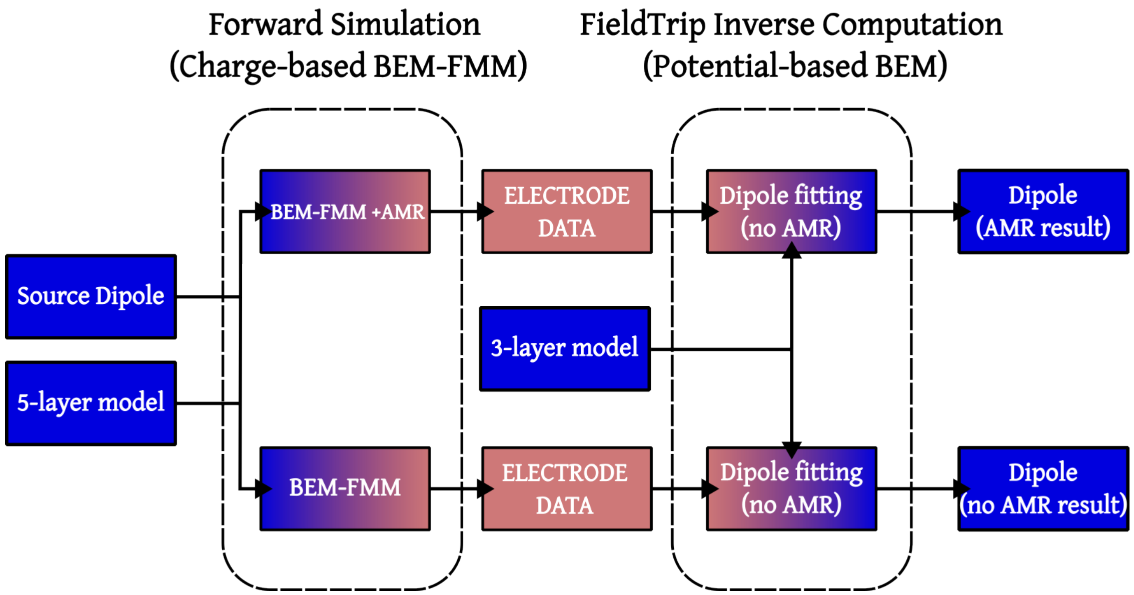

Figure S17: A flowchart for the performance study of 3-layer inverse models using 5-layer forward models. Given a single source dipole and a 5-layer model, we produce two dipole fits using an inverse 3-layer model: one corresponding to an AMR forward solution, and one corresponding to a non-AMR one.
